# Dissociation of inositol 1,4,5-trisphosphate from IP_3_ receptors contributes to termination of Ca^2+^ puffs

**DOI:** 10.1016/j.jbc.2023.102871

**Published:** 2023-01-05

**Authors:** Holly A. Smith, Colin W. Taylor

**Affiliations:** Department of Pharmacology, University of Cambridge, Cambridge, United Kingdom

**Keywords:** calcium intracellular release, calcium imaging, cell signaling, ER, inositol 1,4,5-trisphosphate receptor, Ab-IP_3_R3, IP_3_R3-selective antibody, BSA, bovine serum albumin, [Ca^2+^]_c_, cytosolic free Ca^2+^ concentration, HBS, HEPES-buffered saline, HEK, human embryonic kidney, HEK-3KO, HEK cell with no IP_3_Rs, IBC, IP_3_-binding core, IP_3_, inositol 1,4,5-trisphosphate, IP_3_R, IP_3_ receptor, ROI, region of interest, ROI^puffs^, ROI from which Ca^2+^ puffs were recorded, TIRF, total internal reflection fluorescence

## Abstract

Ca^2+^ puffs are brief, localized Ca^2+^ signals evoked by physiological stimuli that arise from the coordinated opening of a few clustered inositol 1,4,5-trisphosphate receptors (IP_3_Rs). However, the mechanisms that control the amplitude and termination of Ca^2+^ puffs are unresolved. To address these issues, we expressed SNAP-tagged IP_3_R3 in HEK cells without endogenous IP_3_Rs and used total internal reflection fluorescence microscopy to visualize the subcellular distribution of IP_3_Rs and the Ca^2+^ puffs that they evoke. We first confirmed that SNAP-IP_3_R3 were reliably identified and that they evoked normal Ca^2+^ puffs after photolysis of a caged analog of IP_3_. We show that increased IP_3_R expression caused cells to assemble more IP_3_R clusters, each of which contained more IP_3_Rs, but the mean amplitude of Ca^2+^ puffs (indicative of the number of open IP_3_Rs) was unaltered. We thus suggest that functional interactions between IP_3_Rs constrain the number of active IP_3_Rs within a cluster. Furthermore, Ca^2+^ puffs evoked by IP_3_R with reduced affinity for IP_3_ had undiminished amplitude, but the puffs decayed more quickly. The selective effect of reducing IP_3_ affinity on the decay times of Ca^2+^ puffs was not mimicked by exposing normal IP_3_R to a lower concentration of IP_3_. We conclude that distinct mechanisms constrain recruitment of IP_3_Rs during the rising phase of a Ca^2+^ puff and closure of IP_3_Rs during the falling phase, and that only the latter is affected by the rate of IP_3_ dissociation.

Intracellular Ca^2+^ signals regulate many cellular processes. Most Ca^2+^ signals are initiated by inositol 1,4,5-trisphosphate (IP_3_) produced when cell-surface receptors stimulate phospholipase C. IP_3_ binds to the four subunits of a tetrameric IP_3_ receptor (IP_3_R), priming it to bind Ca^2+^, and triggering the opening of an intrinsic Ca^2+^-permeable channel, through which Ca^2+^ flows rapidly from the lumen of the endoplasmic reticulum to the cytosol (1, 2, 3). Ca^2+^ puffs are evoked by low stimulus intensities, similar to those likely to occur under physiological conditions. These Ca^2+^ puffs are brief, localized increases in cytosolic free Ca^2+^ concentration ([Ca^2+^]_c_) that arise from the coordinated opening of a few channels within a cluster (4, 5, 6, 7). Compelling evidence suggests that the amplitude of a Ca^2+^ puff reports the number of open IP_3_Rs. This includes evidence that the amplitudes of steps within the falling phase of a Ca^2+^ puff match those of the very smallest events (‘Ca^2+^ blips’), which report the opening of a single IP_3_R (4). The coordinated openings of IP_3_Rs are thought, at least in part, to arise from costimulation of all IP_3_Rs by IP_3_ and Ca^2+^ (1). An additional, but essential, level of regulation is provided by Kras-induced actin-binding protein (KRAP), which licenses clustered IP_3_Rs to respond to IP_3_ (8).

Most IP_3_Rs within a cell are mobile, but Ca^2+^ puffs arise preferentially from immobile clusters of IP_3_Rs which are tethered near to endoplasmic reticulum-plasma membrane junctions by KRAP (7, 8). These subcellular sites are the same whether Ca^2+^ puffs are evoked by IP_3_ produced by endogenous signaling pathways or by photolysis of a caged analog of IP_3_ (ci-IP_3_) (6, 9). Ca^2+^ puffs may both allow local regulation of Ca^2+^ sensors and contribute to the genesis of global Ca^2+^ signals, although the role of Ca^2+^ puffs in the latter is unresolved (10).

Ca^2+^-induced Ca^2+^-release between IP_3_Rs is potentially explosive, but the mechanisms that terminate the activity of Ca^2+^ puffs are incompletely understood. Delayed feedback inhibition of IP_3_Rs by substantial local increases in [Ca^2+^]_c_ probably contributes (11, 12, 13, 14), but it may not be the only mechanism. Evidence for coupled closing of IP_3_Rs during the falling phase of a Ca^2+^ puff (15) and conflicting evidence implicating luminal Ca^2+^ in the regulation of IP_3_Rs (16, 17) suggest additional factors that may contribute to termination of Ca^2+^ puffs. The effect of cytosolic Ca^2+^ on IP_3_Rs is determined by whether they have IP_3_ bound (1). The simplest scheme suggests that IP_3_ binding allows Ca^2+^ to stimulate channel opening, while Ca^2+^ inhibits IP_3_Rs without IP_3_ bound (18, 19, 20). This scheme suggests that if local increases in [Ca^2+^]_c_ contribute to terminating Ca^2+^ puffs, it may be necessary for IP_3_ to first dissociate from at least one IP_3_R subunit; in that case, the rate of IP_3_ dissociation may be an important determinant of how quickly Ca^2+^ puffs terminate. Furthermore, we might expect coupled closing and inhibition by local increases in [Ca^2+^]_c_ to be influenced by the density of IP_3_Rs within the clusters that evoke Ca^2+^ puffs. These considerations prompted our analyses of the effects of varying IP_3_R expression and IP_3_ affinity on Ca^2+^ puffs.

All three subtypes of IP_3_R evoke similar Ca^2+^ puffs (21, 22), but IP_3_R3 offers advantages for experimental analyses. Plasmids encoding IP_3_R3 are easy to manipulate, there is an excellent IP_3_R3-selective antibody, and there are high-resolution structures of IP_3_R3 in different states (23, 24, 25). We therefore chose human IP_3_R3 for our analyses of Ca^2+^ puffs. By expressing SNAP-tagged IP_3_R3 in cells devoid of native IP_3_R, we were able to visualize both IP_3_Rs and the Ca^2+^ signals they evoke. Our results establish that functional interactions between IP_3_Rs constrain their recruitment during the rising phase of a Ca^2+^ puff and that IP_3_ dissociation from the IP_3_R contributes to termination of each Ca^2+^ puff.

## Results

### SNAP-tagged IP_3_R3 are reliably identified and evoke normal Ca^2+^ puffs

**Figure 1:**
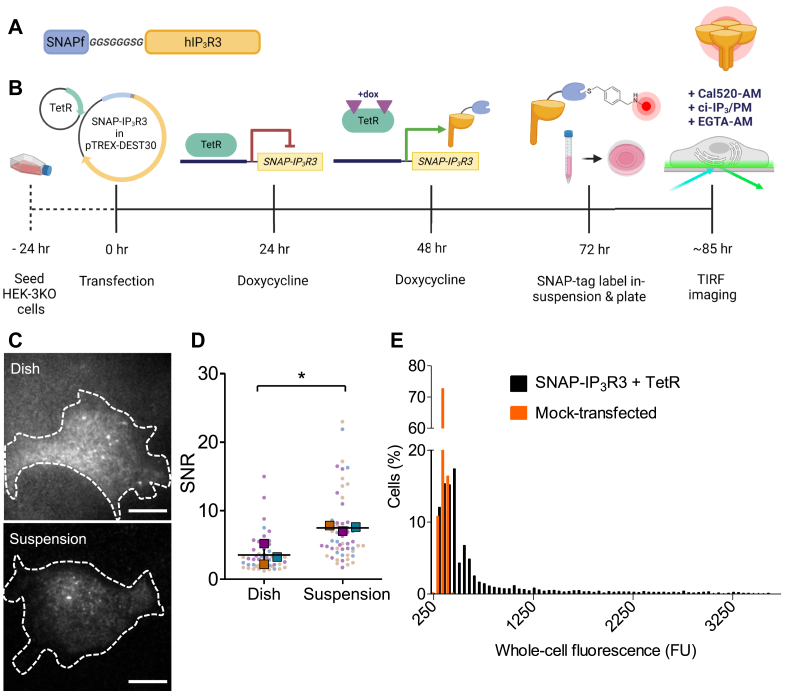
**Controlled expression and visualization of SNAP-IP**_**3**_**R3.***A*, the SNAP-IP_3_R3 construct. A fast-labeling SNAP-tag (SNAPf) is attached to the N-terminus of human IP_3_R3 (hIP_3_R3) *via* a short peptide linker. *B*, expression and labeling of SNAP-IP_3_R3. HEK-3KO cells are transiently transfected with plasmids encoding TetR and SNAP-IP_3_R3 under a tetracycline-inducible CMV promoter. Addition of doxycycline (1 μg/ml, 48 h) then derepresses expression of SNAP-IP_3_R3. HEK-SNAP-IP_3_R3 cells are irreversibly labeled in suspension with a fluorescent SNAP-tag substrate (SNAP-Cell 647-SiR, 1 μM) and plated onto imaging dishes before noninvasive loading with Cal520-AM, ci-IP_3_/PM, and EGTA-AM for TIRF imaging of Ca^2+^ puffs evoked by photolysis of ci-IP_3_. *C*, TIRF images of HEK-SNAP-IP_3_R3 cells labeled with SNAP-Cell 647-SiR (1 μM) in the imaging dish according to the manufacturer’s instructions or in-suspension (see Experimental procedures) and recorded under identical conditions. Scale bars represent 10 μm. *D*, signal-to-noise ratio (SNR) of intracellular/extracellular fluorescence measured from small ROI (5.6 μm^2^) for the two labeling methods shown in *C*. Individual values from 53 cells (points) with mean values from three independent experiments (squares, color-coded) and mean ± S.E.M., ∗*p* < 0.05, unpaired Student’s *t* test. *E*, frequency distributions of whole-cell epifluorescence intensities for HEK-SNAP-IP_3_R3 cells (3892 cells from one experiment) and mock-transfected HEK-3KO cells (2798 cells from one experiment). The results indicate that ∼40% of cells express detectable SNAP-IP_3_R3. FU, fluorescence unit. HEK-3KO cell, HEK cell with no IP_3_Rs; HEK, human embryonic kidney; IP_3_R, inositol 1,4,5-trisphosphate receptor; ROI, region of interest; TIRF, total internal reflection fluorescence.

**Figure 2:**
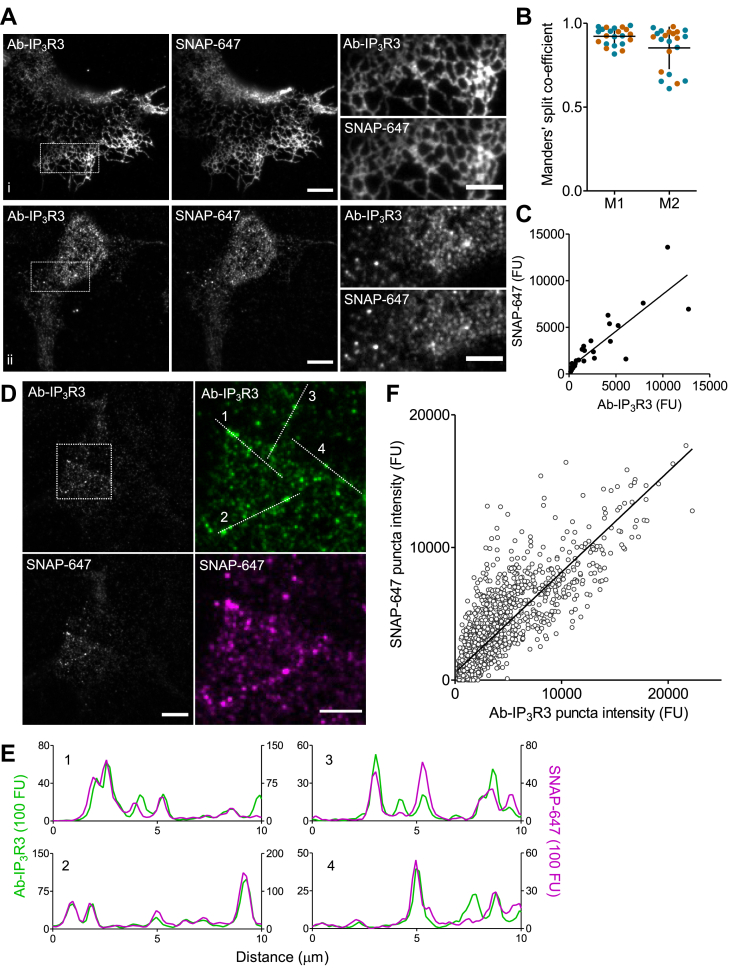
**SNAP-tag labeling reliably reports IP**_**3**_**R distribution.***A*, TIRF images of HEK-SNAP-IP_3_R3 cells immunostained with Ab-IP_3_R3 and labeled with SNAP-Cell 647-SiR (SNAP-647) at high (i) and low (ii) levels of SNAP-IP_3_R3 expression. Scale bars represent 10 μm, 5 μm in enlargements of boxed areas. *B*, Manders’ split coefficient values for colocalization of Ab-IP_3_R3 fluorescence with SNAP-647 fluorescence (M1) and vice versa (M2) for cells expressing different amounts of IP_3_R3 (see panel *C*). Individual values from 22 cells from two independent analyses (color-coded) and mean ± S.D. *C*, relationship between whole-cell fluorescence intensities from the entire TIRF footprint for SNAP-647 and Ab-IP_3_R3 staining. Pearson correlation coefficient *r* = 0.87, *p* < 0.0001, *n* = 34 cells from two independent analyses. *D*, TIRF images of a HEK-SNAP-IP_3_R3 cell showing punctate Ab-IP_3_R3 and SNAP-647 fluorescence. Scale bars represent 10 μm, 5 μm in pseudo-colored enlargements of boxed area. *E*, fluorescence intensity profiles along the lines (1, 2, 3, 4) shown in *D*. *F*, relationship between fluorescence intensities of individual SNAP-647 and Ab-IP_3_R3 puncta. 2310 puncta from 10 cells from two independent analyses. Pearson correlation coefficient *r* = 0.85, *p* < 0.0001. Ab-IP_3_R3, IP_3_R3-selective antibody; HEK, human embryonic kidney; IP_3_R, inositol 1,4,5-trisphosphate receptor; TIRF, total internal reflection fluorescence.

**Figure 3:**
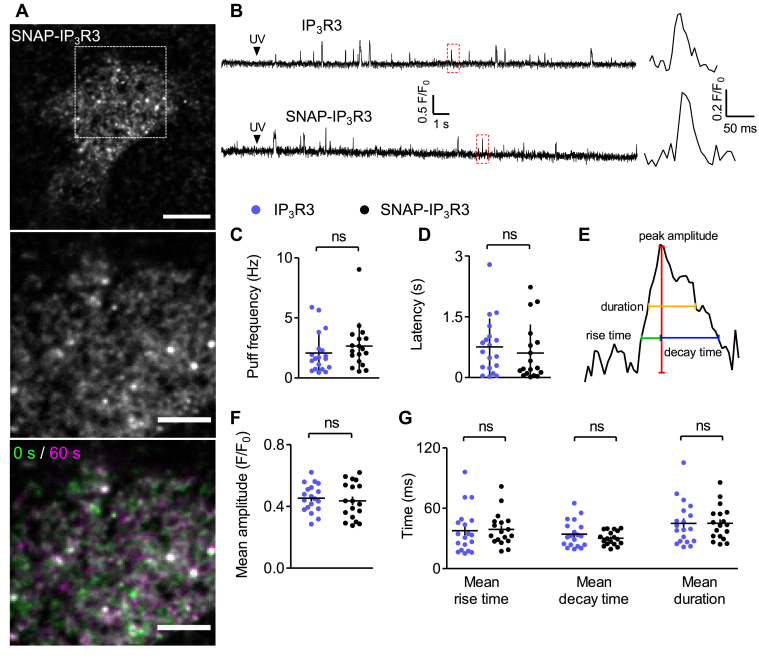
**Similar Ca**^**2+**^**puffs are evoked by endogenous IP**_**3**_**R3 and SNAP-IP**_**3**_**R3.***A*, typical TIRF image of SNAP-IP_3_R3 in a live cell selected for analysis because the IP_3_R expression reveals mobile and immobile puncta typical of native expression (7) without the clear delineation of reticular ER that occurs with over-expression. Boxed area (enlarged below) shows the region (19.2 × 19.2 μm) from which Ca^2+^ puffs were recorded (ROI^puffs^). The time-overlay of images captured at 0 s (*green*) and 60 s (*magenta*) show immobile SNAP-IP_3_R3 puncta (*white*). Scale bars represent 10 μm, 5 μm in enlargements. *B*, fluorescence traces show Ca^2+^ puffs evoked by photolysis of ci-IP_3_ (250-ms UV flash, arrows) in a HEK-IP_3_R3 or HEK-SNAP-IP_3_R3 cell. Cal520 fluorescence (F) was recorded in TIRF from a small region (1.76 × 1.76 μm), wherein Ca^2+^ puffs occurred repeatedly and is expressed relative to fluorescence recorded before the UV flash (F_0_). Enlargements of boxed areas show individual Ca^2+^ puffs. *C* and *D*, frequency and latency (time from UV flash to first Ca^2+^ puff) of Ca^2+^ puffs evoked through SNAP-IP_3_R3 and endogenous IP_3_R3. Results show individual cells and mean ± S.D. *E*, measured properties of Ca^2+^ puffs (F/F_0_): amplitude (baseline to peak), rise time (20% to 100% peak amplitude), decay time (100% to 20% peak amplitude), and duration (at half-maximal amplitude). *F* and *G*, mean amplitudes (*F*) and mean kinetic properties (*G*) of Ca^2+^ puffs evoked by IP_3_R3 and SNAP-IP_3_R3. Results show mean values from single cells and mean ± S.E.M. Results (*C*, *D*, *F*, and *G*) are from 19 cells from five independent experiments for IP_3_R3 and from 18 cells from three independent experiments for SNAP-IP_3_R3. ns *p* > 0.05, unpaired Student’s *t* test. HEK, human embryonic kidney; IP_3_R, inositol 1,4,5-trisphosphate receptor; ROI^puffs^, ROI from which Ca^2+^ puffs were recorded; TIRF, total internal reflection fluorescence.

To define the subcellular distribution of IP_3_R3 while measuring the Ca^2+^ puffs they evoke, human IP_3_R3 fused to a fast-labeling SNAP-tag (SNAP-IP_3_R3, Fig. 1*A*) was expressed in human embryonic kidney (HEK) cells lacking endogenous IP_3_Rs (HEK-3KO cells) (2). We used an N-terminal tag because it does not disrupt IP_3_R function (7) and a SNAP-tag (19.4 kDa; smaller than GFP) that allows versatile covalent labeling with fluorophores (Fig. 1*B*) (26). Our use of HEK-3KO cells (2) and transient expression of SNAP-IP_3_R3 under control of an inducible promoter ensured that functional responses were entirely mediated by SNAP-IP_3_R3 homotetrameric channels expressed at appropriate levels (Fig. 1*B*). Our optimized methods, which required labeling of cells in suspension before plating for analyses using fluorescence microscopy (Fig. 1, *A–D*), allowed SNAP-IP_3_R3 to be identified in cells, most of which expressed IP_3_R3 at modest levels (Fig. 1*E*) and with the punctate distribution of IP_3_R clusters typical of native IP_3_Rs (Fig. 2*Aii* and see Fig. 3*A*) (7).

We confirmed the specificity of an antibody to IP_3_R3 (Ab-IP_3_R3) (27) in immunocytochemical analyses by demonstrating that Ab-IP_3_R3 stains puncta in HEK-SNAP-IP_3_R3 cells, but not in HEK-3KO cells (Fig. S1). HEK-SNAP-IP_3_R3 cells labeled with SNAP-Cell 647-SiR (hereafter, SNAP-647) were immunostained with Ab-IP_3_R3 and imaged using total internal reflection fluorescence (TIRF) microscopy. The results demonstrate colocalization of immunostaining and SNAP-647 fluorescence (Fig. 2, *A*, *B, D*, and *E*), a tight linear correlation between the intensities of whole-cell immunostaining and SNAP-647 fluorescence (Fig. 2*C*), and a linear correlation for individual puncta between Ab-IP_3_R3 and SNAP-647 fluorescence intensities (Figs. 2*F* and S2). The results so far (Figs. 1 and 2) demonstrate that transfection of HEK-3KO cells with SNAP-IP_3_R3 and subsequent labeling with SNAP-647 allows near-native levels of IP_3_R expression and reliable detection of all IP_3_R3.

We used TIRF microscopy to compare Ca^2+^ puffs evoked by photolysis of ci-IP_3_ in HEK-IP_3_R3 and HEK-SNAP-IP_3_R3 cells. Since the latter were transiently transfected (Fig. 1*B*), individual cells differed in their expression of SNAP-IP_3_R3 (Fig. 1*E*), we therefore selected cells in which the distribution of IP_3_R resembled that observed in HeLa cells with tagged endogenous IP_3_R (7), namely cells with comparatively low SNAP-647 fluorescence intensities in which most IP_3_R puncta were mobile and a smaller fraction were immobile (Fig. 3*A*). The results demonstrate that Ca^2+^ puffs occurred with indistinguishable frequencies and after similar latencies in the two cell types (Fig. 3, *B–D*). Furthermore, the properties of individual Ca^2+^ puffs (mean amplitudes, rise times, decay times, and durations) were also indistinguishable for HEK-IP_3_R3 and HEK-SNAP-IP_3_R3 cells (Figs. 3, *E*–*G* and S3).

Our results establish that HEK-SNAP-IP_3_R3 cells after labeling with SNAP-647 allow reliable identification of all IP_3_Rs within a cell and unperturbed IP_3_-evoked Ca^2+^ puffs. We use these cells to explore the effects of varying IP_3_R expression and the rate of IP_3_ dissociation from IP_3_R on the properties of Ca^2+^ puffs.

### Increased IP_3_R expression increases the frequency of Ca^2+^ puffs and the size of IP_3_R clusters without affecting the properties of Ca^2+^ puffs

**Figure 4:**
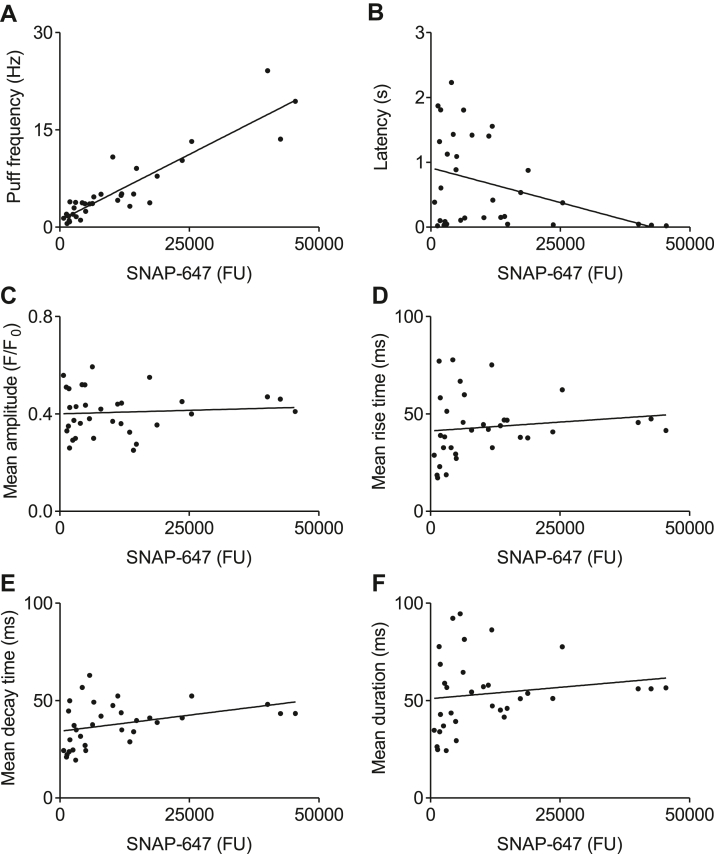
**Ca**^**2+**^**puffs are more frequent in cells over-expressing IP**_**3**_**R, but the properties of individual Ca**^**2+**^**puffs are unaffected.***A*, relationship between SNAP-647 fluorescence intensity (recorded from the 19.2 × 19.2 μm region where Ca^2+^ puffs were recorded, ROI^puffs^) and the frequency of Ca^2+^ puffs evoked by photolysis (250 ms) of ci-IP_3_. Regression line shows a positive correlation between the frequency of Ca^2+^ puffs and SNAP-IP_3_R3 expression. Pearson correlation coefficient *r* = 0.91, *p* < 0.0001. Results show 33 cells from five experiments. *B*, relationship between SNAP-IP_3_R3 expression and latency to the first Ca^2+^ puff detected after the UV flash; *r* = −0.37, *p* < 0.05. *C*–*F*, relationships between SNAP-IP_3_R3 expression and mean puff amplitude (*C*), mean rise time (*D*), mean decay time (*E*), and mean duration (*F*) of individual Ca^2+^ puffs. Results show 33 cells from five experiments. FU, fluorescence unit; IP_3_R, inositol 1,4,5-trisphosphate receptor; ROI^puffs^, ROI from which Ca^2+^ puffs were recorded.

We used SNAP-647 fluorescence intensity measured from the TIRF footprint of the region of interest (ROI) from which Ca^2+^ puffs were recorded (ROI^puffs^, 19.2 × 19.2 μm) to report SNAP-IP_3_R3 expression in individual HEK-SNAP-IP_3_R3 cells and compared it with the properties of the Ca^2+^ puffs evoked by photolysis of ci-IP_3_. The results demonstrate that over about a 60-fold range of expression there is a positive correlation between SNAP-IP_3_R3 expression and the frequency of Ca^2+^ puffs (Fig. 4*A*): Ca^2+^ puffs are more frequent in cells with more IP_3_Rs. The latency to the first detected Ca^2+^ puff after the photolysis flash decreased as SNAP-IP_3_R3 expression increased (Fig. 4*B*). However, the mean properties of individual Ca^2+^ puffs (amplitude, rise and decay times, and durations) were similar at all levels of SNAP-IP_3_R3 expression (Fig. 4, *C*–*F*).

**Figure 5:**
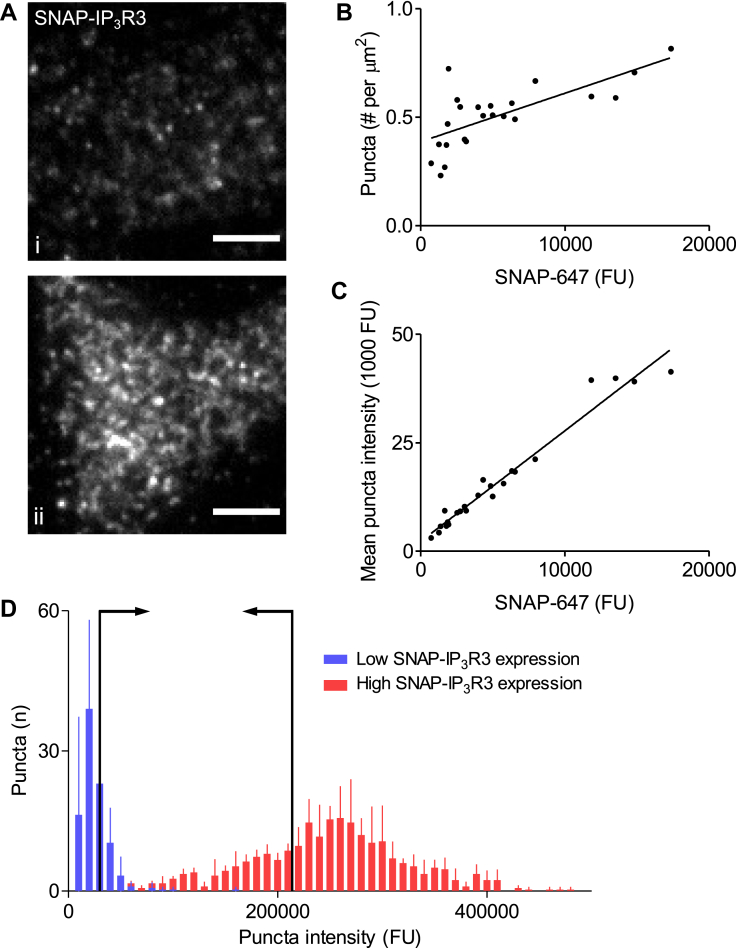
**IP**_**3**_**R clusters are more abundant and contain more IP**_**3**_**Rswith increased expression of IP**_**3**_**R.***A*, live-cell TIRF images of HEK-SNAP-IP_3_R3 cells expressing low (i) or high (ii) levels of SNAP-IP_3_R3. Scale bars represent 5 μm. Images were captured under identical conditions and with identical display values. *B* and *C*, relationships between overall SNAP-IP_3_R3 expression (SNAP-647 fluorescence intensity measured from ROI^puffs^) and the number of SNAP-IP_3_R3 puncta (*B,* Pearson correlation coefficient *r* = 0.71, *p* < 0.001) and mean fluorescence intensity of individual puncta (*C, r* = 0.98, *p* < 0.0001). Results show 23 cells from four experiments. Analysis was restricted to cells in which puncta could be resolved (SNAP-647 fluorescence <20,000 FU). *D*, fluorescence intensity distributions for individual puncta from cells expressing the lowest (*blue*) and highest (*red*) levels of SNAP-IP_3_R3 (SNAP-647 fluorescence = 1133 ± 350 and 15,226 ± 1950 FU, respectively; n = 3 cells for each category). Results show number of puncta identified within ROI^puffs^ (mean ± S.D., n = 3). Arrows indicate the brightest 30% of puncta in cells with fewest IP_3_Rs and the dimmest 30% in cells with most IP_3_Rs (about 30% of puncta are expected to be active Ca^2+^ puff sites, Fig. S5*C*). The lack of overlap between these two populations indicates that Ca^2+^ puffs in cells with most IP_3_Rs are evoked by clusters that contain more IP_3_Rs. FU, fluorescence unit; HEK, human embryonic kidney; IP_3_R, inositol 1,4,5-trisphosphate receptor; ROI^puffs^, ROI from which Ca^2+^ puffs were recorded; TIRF, total internal reflection fluorescence.

We next asked how IP_3_R are distributed within cells expressing different numbers of IP_3_R. We restricted this analysis to HEK-SNAP-IP_3_R3 cells in which the SNAP-647 fluorescence intensity measured from ROI^puffs^ was <20,000 fluorescence units because at higher levels of expression, it was impossible to reliably distinguish individual puncta. The results demonstrate that both the number of SNAP-IP_3_R3 puncta (Fig. 5*A* and *B*) and their mean fluorescence intensity (Fig. 5, *A* and *C*) were increased in cells expressing more SNAP-IP_3_R3. The rightward shifts in the fluorescence intensity distributions for individual puncta as IP_3_R expression increased indicates that when cells express more IP_3_Rs, most puncta contain more IP_3_Rs (Fig. S4). This analysis indicates that clusters of IP_3_Rs within the TIRF field are more abundant and each cluster includes more IP_3_Rs in cells with more IP_3_Rs.

These results demonstrate that across a wide range of SNAP-IP_3_R3 expression (∼60-fold), the properties of individual Ca^2+^ puffs, including their mean amplitudes, are preserved, but they occur after shorter latencies and they are more frequent in cells with more IP_3_Rs. Since only a fraction of IP_3_R puncta are licensed to respond (7), we had to consider whether in cells with most IP_3_Rs, a few puncta with a normal complement of IP_3_R might lurk beneath the substantial increase in the average number of IP_3_Rs per punctum and thereby explain the unaltered properties of individual Ca^2+^ puffs. To address this issue, we recognize that about 30% of IP_3_R puncta are competent to evoke Ca^2+^ puffs (Fig. S5) (7) and therefore compared the brightest 30% of puncta in cells with the fewest SNAP-IP_3_R3 to the dimmest puncta in cells with most SNAP-IP_3_R3. The results demonstrate that in cells with most IP_3_Rs, the number of puncta with fluorescence intensities comparable to the brightest puncta of cells with fewest IP_3_Rs is far too low to underlie the observed number of sites at which Ca^2+^ puffs occur (Fig. 5*D*). This analysis demonstrates that in cells expressing many IP_3_Rs, puncta that contain more IP_3_Rs evoke Ca^2+^ puffs of unaltered amplitude.

We conclude that the amplitude of Ca^2+^ puffs, indicating the number of open IP_3_Rs (4, 5, 6, 7), is similar in cells expressing very different numbers of IP_3_Rs. Since IP_3_R clusters are larger in cells with more IP_3_Rs, functional interactions between IP_3_Rs must determine the number of IP_3_Rs that contribute to a Ca^2+^ puff. This conclusion prompted further analysis of the mechanisms that might terminate the activity of IP_3_R during a Ca^2+^ puff.

### IP_3_R with reduced affinity for IP_3_ generate briefer Ca^2+^ puffs

**Figure 6:**
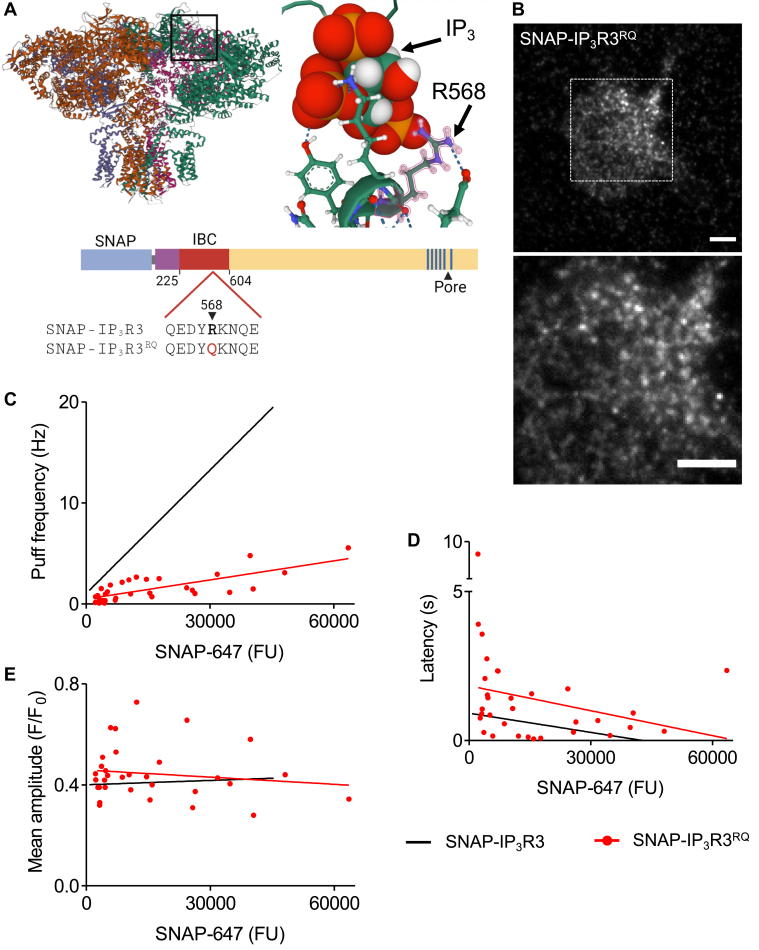
**Ca**^**2+**^**puffs are less frequent in cells expressing IP**_**3**_**R with reduced affinity for IP**_**3**_**, but their amplitude is unaffected.***A*, structure of human IP_3_R3 showing R568 in the IP_3_-binding core (IBC) coordinating IP_3_ (Protein Data Bank, 6DQN) (23). Mutation of R568Q within the IBC of SNAP-IP_3_R3 reduces its affinity for IP_3_. *B*, live-cell TIRF image of a HEK-SNAP-IP_3_R3^RQ^ cell, enlarged in lower panel. Scale bars represent 5 μm. The punctate distribution is similar to that of SNAP-IP_3_R3 (Figs. 1*C*, 2, *A* and *D*, and 3*A*). *C–E*, relationships between SNAP-IP_3_R3^RQ^ expression (from ROI^puffs^) and the frequency of Ca^2+^ puffs evoked by photolysis of ci-IP_3_ (*C*) (*r* = 0.75, *p* < 0.0001), latency to the first Ca^2+^ puff after the photolysis flash (*D*), and the mean amplitude of Ca^2+^ puffs (*E*) (slopes not significantly different from 0). Results are from 32 cells from five experiments. Linear regression analyses for SNAP-IP_3_R3 (from Fig. 4, *A–C*) show that frequencies are lower, latencies are longer, and mean amplitudes of Ca^2+^ puffs are unaffected in SNAP-IP_3_R3^RQ^ cells. FU, fluorescence unit; HEK, human embryonic kidney; IP_3_R, inositol 1,4,5-trisphosphate receptor; ROI^puffs^, ROI from which Ca^2+^ puffs were recorded; TIRF, total internal reflection fluorescence.

Since the mechanisms that terminate Ca^2+^ puffs are unresolved (15, 28), we asked whether IP_3_ dissociation from IP_3_R contributes to termination. A point mutation (R568Q) within the conserved IP_3_-binding core (IBC) of IP_3_R1 causes a 9-fold reduction of its affinity for IP_3_ (29). We introduced the same mutation into SNAP-IP_3_R3 and used it to explore the effects of increasing the rate of IP_3_ dissociation on Ca^2+^ puffs (Fig. 6*A*). We confirmed that SNAP-IP_3_R3^RQ^ had the same subcellular distribution as SNAP-IP_3_R3 (Fig. 6*B*). Since the IBC is highly conserved in all IP_3_R subtypes and each IBC binds IP_3_ with the same affinity (30), we expect the IP_3_R3^RQ^ mutation to exactly replicate the 9-fold decrease in affinity reported for IP_3_R1^RQ^ (29). The assumption is supported by evidence that the frequency of Ca^2+^ puffs evoked by photolysis of ci-IP_3_ is reduced in cells expressing IP_3_R3^RQ^ (Fig. 6*C*).

We expected that reducing the affinity of an IP_3_R for IP_3_ while maintaining the submaximal concentration of i-IP_3_ delivered after the UV flash to reduce the number of IP_3_R occupied by i-IP_3_ and so reduce the frequency of Ca^2+^ puffs and increase the latency before the first observed Ca^2+^ puff. Our results confirm these expectations. As with SNAP-IP_3_R3, the frequency of Ca^2+^ puffs increased with increased expression of SNAP-IP_3_R_3_^RQ^, the latencies shortened with increased expression, and the mean amplitude of individual Ca^2+^ puffs was unaffected by the overall level of SNAP-IP_3_R3^RQ^ expression (Fig. 6, *C–E*). However, across a wide range of IP_3_R3 expression levels, the frequencies of Ca^2+^ puffs were lower, and the latencies were longer for SNAP-IP_3_R3^RQ^ than for SNAP-IP_3_R3 (Fig. 6, *C* and *D*).

**Figure 7:**
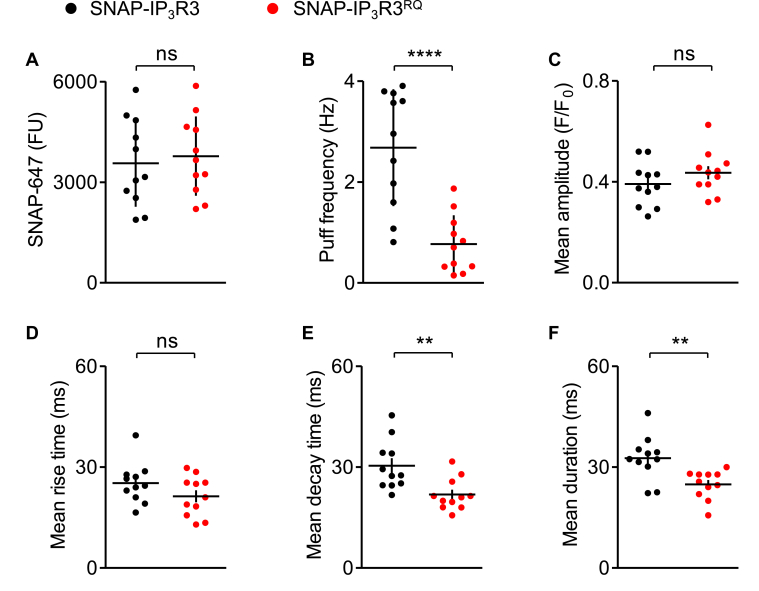
**IP**_**3**_**R with reduced affinity for IP**_**3**_**evoke Ca**^**2+**^**puffs that terminate more quickly.***A*, SNAP-647 fluorescence intensity measured from ROI^puffs^ for SNAP-IP_3_R3 and SNAP-IP_3_R3^RQ^ cells selected for analyses of kinetics of Ca^2+^ puffs. Selection criteria (1800–5900 FU) were chosen to include cells with near-endogenous expression of IP_3_R3. *B* and *C*, frequency (*B*) and mean amplitude (*C*) of Ca^2+^ puffs evoked by photolysis of ci-IP_3_ in cells that satisfied the selection criteria. *D*–*F*, mean rise times (*D*), decay times (*E*), and durations (*F*) of Ca^2+^ puffs in the analyzed cells, where Ca^2+^ puffs with ‘square’ temporal profiles were excluded (Fig. S6, *A* and *B*). Results show mean ± S.D. (*A* and *B*) or S.E.M. (*C*–*F*) for 11 cells from four experiments for SNAP-IP_3_R3 or 11 cells from five experiments for SNAP-IP_3_R3^RQ^. ns *p* > 0.05, ∗∗*p* < 0.01, ∗∗∗∗*p* < 0.0001, unpaired Student’s *t* test. FU, fluorescence unit; IP_3_R, inositol 1,4,5-trisphosphate receptor; ROI^puffs^, ROI from which Ca^2+^ puffs were recorded.

To allow direct comparisons of cells expressing SNAP-IP_3_R3 and SNAP-IP_3_R3^RQ^, we pooled data from cells in which the SNAP-647 fluorescence intensity measured from ROI^puffs^ fell within a defined range (1800–5900 FU), which coincided with cells in which the subcellular distribution of IP_3_R3 resembled that of native IP_3_Rs. The pooled data confirmed that the average level of IP_3_R3 expression (Fig. 7*A*) and mean puff amplitude (Fig. 7*C*) were indistinguishable for HEK-SNAP-IP_3_R3 and HEK-SNAP-IP_3_R3^RQ^ cells, but the frequency of Ca^2+^ puffs was significantly reduced in the HEK-SNAP-IP_3_R3^RQ^ cells (Fig. 7*B*).

We next compared the kinetic properties of individual Ca^2+^ puffs in cells expressing SNAP-IP_3_R3 or SNAP-IP_3_R3^RQ^. Automated analysis of the rise times of Ca^2+^ puffs is distorted when Ca^2+^ puffs have a sustained plateau phase (‘square’ puffs) (15) because stochastic fluctuations in fluorescence during the plateau can delay the peak amplitude and thereby erratically extend the computed rise time (Fig. S6*A*). We therefore excluded ‘square’ Ca^2+^ puffs from our analyses of the kinetics of individual Ca^2+^ puffs. The exclusion rate was similar (∼18%) for analyses of SNAP-IP_3_R3 and SNAP-IP_3_R3^RQ^ (Fig. S6*B*), confirming that it did not bias our analyses. We note in passing that although we have not specifically addressed the mechanisms underlying ‘square’ Ca^2+^ puffs, their similar abundance in cells expressing IP_3_R3 and IP_3_R3^RQ^ indicates that ‘square’ Ca^2+^ puffs are not selectively affected by changes in IP_3_R affinity. Rise times were indistinguishable in cells expressing SNAP-IP_3_R3 and SNAP-IP_3_R3^RQ^ (Fig. 7*D*), but decay times and the duration of Ca^2+^ puffs were significantly shorter for SNAP-IP_3_R3^RQ^ (Figs. 7, *E* and *F* and 6, *C–E*).

**Figure 8:**
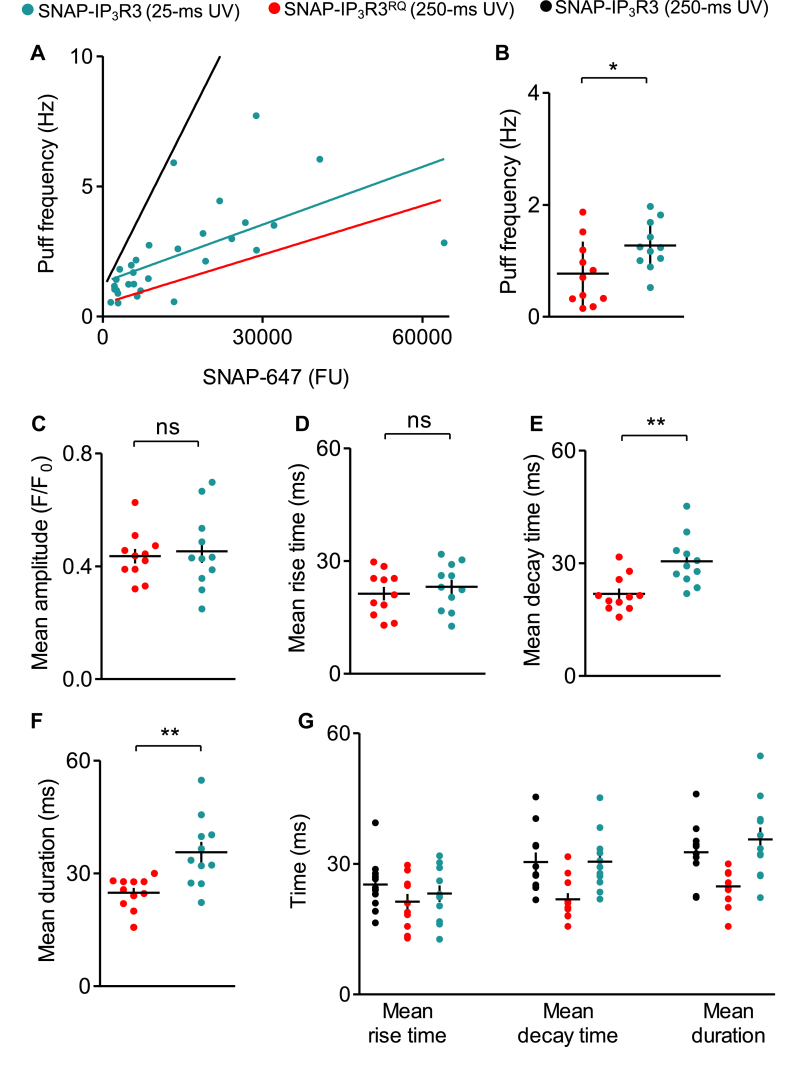
**Shortened Ca**^**2+**^**puffs from IP**_**3**_**R3**^**RQ**^**are not a consequence of reduced occupancy of IP**_**3**_**R by IP**_**3**_**.***A*, relationship between SNAP-IP_3_R3 expression (SNAP-647 fluorescence intensity measured from ROI^puffs^) and the frequency of Ca^2+^ puffs recorded after photolysis of ci-IP_3_ from HEK-SNAP-IP_3_R3 cells stimulated with a brief UV flash (25 ms). Results are from 30 cells from four experiments; Pearson correlation coefficient *r* = 0.60, *p* < 0.001. Regression lines (from Figs. 4*A* and 6*C*) show results from similar analyses of SNAP-IP_3_R3 and SNAP-IP_3_R3^RQ^ stimulated with a 10-fold longer flash (250 ms). *B*–*F*, frequency of Ca^2+^ puffs (*B*), and mean amplitude (*C*), rise time (*D*), decay time (*E*), and duration (*F*) for individual Ca^2+^ puffs. Results (mean ± S.E.M., or S.D. for *B*) are from 11 cells from four experiments for SNAP-IP_3_R3 (25-ms UV) or 11 cells from five experiments for SNAP-IP_3_R3^RQ^ (250-ms UV). Selection criteria (SNAP-647 fluorescence intensity of 1800–5900 FU, measured from ROI^puffs^) were chosen to include cells with near-endogenous expression of IP_3_R3 (*B*–*F*), and square Ca^2+^ puffs were excluded from analyses of kinetics (*D*–*F*). ns *p* > 0.05, ∗*p* < 0.05, ∗∗*p* < 0.01, unpaired Student’s *t* test. *G*, overall summary (individual values, mean ± S.E.M.) shows the kinetic properties of Ca^2+^ puffs evoked by flash photolysis of ci-IP_3_ with the indicated flash durations for HEK-SNAP-IP_3_R3 or HEK-SNAP-IP_3_R3^RQ^ cells (compiled from Figs. 7, *D*–*F* and 8, *D*–*F*). FU, fluorescence unit; HEK, human embryonic kidney; IP_3_R, inositol 1,4,5-trisphosphate receptor; ROI^puffs^, ROI from which Ca^2+^ puffs were recorded.

If the effects of reducing the affinity of the IP_3_R for IP_3_ were solely attributable to reduced occupancy of IP_3_R by IP_3_, we would expect Ca^2+^ puffs evoked by a 9 to 10-fold lower concentration of i-IP_3_ through normal IP_3_R to have the same properties as Ca^2+^ puffs evoked by IP_3_R^RQ^. We therefore compared Ca^2+^ puffs evoked by photolysis of ci-IP_3_ in HEK-SNAP-IP_3_R3^RQ^ cells with the usual UV flash duration (250 ms) to those evoked in HEK-SNAP-IP_3_R3 cells exposed to a 10-fold shorter flash (25 ms). The frequency of the Ca^2+^ puffs and the relationship between IP_3_R expression and frequency were similar under the two conditions (Fig. 8, *A* and *B*). These results confirm that for the frequency of Ca^2+^ puffs, a 9 to 10-fold reduction in IP_3_-binding affinity can be compensated by a comparable increase in i-IP_3_ concentration, suggesting that steady-state occupancy of IP_3_R by IP_3_ may determine the frequency of Ca^2+^ puffs. As expected, the amplitudes of individual Ca^2+^ puffs recorded under the two conditions were indistinguishable (Fig. 8*C*). The more important result is that neither the shorter duration of Ca^2+^ puffs nor the faster decay times in cells expressing IP_3_R3^RQ^ were reproduced by exposing IP_3_R3 to a 10-fold lower concentration of i-IP_3_ (Fig. 8, *D*–*F*). Our results show that in cells expressing SNAP-IP_3_R3, all measured properties of individual Ca^2+^ puffs are indistinguishable whether evoked by low concentrations of i-IP_3_ (25-ms flash) or a 10-fold higher concentration (250-ms flash), but the decay times and durations are longer than for IP_3_R with reduced affinity for IP_3_ (SNAP-IP_3_R3^RQ^) (Fig. 8*G*).

## Discussion

We developed methods that allowed homomeric IP_3_R3 to be expressed in HEK cells at levels comparable to expression of endogenous IP_3_R and, by attaching a SNAP-tag to the expressed IP_3_R3, we were able to reveal both the subcellular distribution of IP_3_R3 and the Ca^2+^ puffs they evoke in response to photolysis of ci-IP_3_ (Figs. 1 and 2). The expressed SNAP-IP_3_R3 and native IP_3_R3 evoked indistinguishable Ca^2+^ puffs (Fig. 3).

We established that cells with more IP_3_Rs have more IP_3_R puncta within the TIRF field, each punctum included more IP_3_Rs (Fig. 5), and Ca^2+^ puffs occurred more frequently and after shorter latencies. However, the properties of individual Ca^2+^ puffs were indistinguishable at all levels of IP_3_R expression (Fig. 4). We confirmed, by analysis of the fluorescence intensity distributions of puncta in cells expressing different numbers of IP_3_Rs, that the mean peak amplitude of a Ca^2+^ puff remained constant even as the number of IP_3_Rs within a cluster increased (Figs. 4 and 5). These observations demonstrate that functional interactions between IP_3_Rs within a cluster must constrain the number of IP_3_Rs activated during the rising phase of a Ca^2+^ puff.

Local increases in [Ca^2+^]_c_ are thought to contribute to both rapid recruitment of IP_3_R activity during the rising phase of a Ca^2+^ puff (through costimulation of IP_3_R by Ca^2+^ and IP_3_) and to termination of Ca^2+^ puffs (through delayed inhibition of IP_3_R by Ca^2+^). It is, therefore, relevant that the biphasic effects of [Ca^2+^]_c_ on IP_3_R is itself regulated by IP_3_. We, for example, have suggested that IP_3_ binding to IP_3_R primes them to open by facilitating the binding of Ca^2+^ to a stimulatory site; while in the absence of IP_3_, Ca^2+^ binds to an inhibitory site that prevents channel opening (18, 19, 20). Others suggest variants of this simple scheme that also have IP_3_ regulating the effects of Ca^2+^ on IP_3_R gating (1, 31). The key point is that the rate of IP_3_ dissociation from an active IP_3_R may influence its susceptibility to Ca^2+^ inhibition. We therefore examined the effect of manipulating the rate of IP_3_R dissociation on the properties of Ca^2+^ puffs. We demonstrated that IP_3_R3 with about a 10-fold lower affinity for IP_3_ (SNAP-IP_3_R3^RQ^) evoked Ca^2+^ puffs in response to photolysis of ci-IP_3_. As expected, the Ca^2+^ puffs mediated by SNAP-IP_3_R3^RQ^ were less frequent than those mediated by SNAP-IP_3_R3, consistent with the steady-state occupancy of IP_3_R by IP_3_ determining the number of active IP_3_Rs (Fig. 6). More importantly, while the mean peak amplitudes of the Ca^2+^ puffs were similar for SNAP-IP_3_R3 and SNAP-IP_3_R3^RQ^, indicating similar numbers of open IP_3_Rs, the Ca^2+^ puffs decayed more rapidly for SNAP-IP_3_R3^RQ^ (Fig. 7). Furthermore, the disparity in decay times between SNAP-IP_3_R3 and SNAP-IP_3_R3^RQ^ remained when the intensity of the photolysis flash (and so the intracellular concentration of i-IP_3_) was reduced for SNAP-IP_3_R3 cells to provide matched frequencies of Ca^2+^ puffs in the two cell lines (indicative of comparable IP_3_R occupancies by i-IP_3_) (Fig. 8). These analyses establish that the affinity of an IP_3_R for IP_3_ affects the rate at which Ca^2+^ puffs terminate: reducing the affinity for IP_3_ (increasing the rate of IP_3_ dissociation) causes Ca^2+^ puffs to terminate more quickly. We draw two important conclusions from these data. Firstly, the rate of IP_3_ dissociation from IP_3_R contributes to termination of Ca^2+^ puffs. Various mechanisms might align with this conclusion, but it is consistent with a local increase in [Ca^2+^]_c_ contributing to termination by closing IP_3_R but only after IP_3_ has dissociated from active IP_3_R (19, 20).

Our second conclusion relates to whether the same mechanism might account for the constraint on the number of IP_3_Rs recruited within a cluster during the rising phase of a Ca^2+^ puff and the closure of active IP_3_Rs during the falling phase. The key observation here is that although Ca^2+^ puffs evoked by IP_3_R with reduced affinity for IP_3_ (SNAP-IP_3_R3^RQ^) terminate more quickly, their mean peak amplitude is indistinguishable from that of Ca^2+^ puffs evoked by normal IP_3_Rs (Figs. 7 and 8). This implies that different mechanisms constrain the opening of IP_3_Rs during the rising phase of a Ca^2+^ puff and closure of open IP_3_Rs during the falling phase. A speculative explanation is that during the rising phase of a Ca^2+^ puff, Ca^2+^ released by active IP_3_Rs rapidly inhibits neighboring IP_3_Rs that have not bound IP_3_, while during the falling phase, dissociation of IP_3_ (perhaps from only a single IP_3_R subunit) causes both channel closure and its immediate susceptibility to Ca^2+^ inhibition, which might then prevent reopening.

We conclude that when presented with more IP_3_Rs, cells incorporate more of them into clusters, assemble more IP_3_R clusters, and generate more frequent Ca^2+^ puffs in response to IP_3_, but the properties of individual Ca^2+^ puffs, including their mean peak amplitudes, are unchanged. We suggest that different mechanisms constrain recruitment of IP_3_Rs during the rising phase of a Ca^2+^ puff and cause closure of IP_3_Rs during the falling phase; only the latter is affected by the rate of IP_3_ dissociation.

## Experimental procedures

### Materials

Cal520-AM and Calbryte590-AM were from AAT Bioquest. ci-IP_3_/PM (D-2,3-O-isopropylidene-6-O-(2-nitro-4,5-dimethoxy)benzyl-*myo*-inositol 1,4,5-trisphosphate hexakis(propionoxymethyl) ester) was from SiChem. 35-mm glass-bottom imaging dishes (14-mm micro-well, #1 cover glass) were from Cellvis (IBL Baustoff+Labor GmbH). EGTA-AM, human plasma fibronectin, Pluronic F-127, and doxycycline hydrochloride were from Merck. SNAP-Cell 647-SiR, BsrGI-HF restriction enzyme, Q5 High-Fidelity Master Mix, and Gibson Assembly Master Mix were from New England Biolabs. pcDNA3.2/V5-DEST, pDONR221, pcDNA6/TR and pT-Rex-DEST30 vectors, LR and BP Clonase II enzyme mixes, Pfl23II (a.k.a. BsiWI) and CpoI (a.k.a. RsrII) restriction enzymes, and custom oligonucleotides were from Thermo Fisher Scientific.

Sources of additional materials are provided in relevant sections of Experimental procedures.

### Generation of SNAP-IP_3_R3 constructs

A plasmid encoding human IP_3_R3 was obtained from Harvard PlasmID Database (Clone ID: HsCD00399229, GenBank Accession: BC172406) and cloned from pENTR223 into pcDNA3.2/V5-DEST using LR Gateway cloning. We tagged human IP_3_R3 with a SNAP-tag, a self-labeling enzyme tag based on the human DNA repair protein, O^6^-alkylguanine-DNA-alkyltransferase, which reacts with O^6^-benzylguanine derivatives conjugated to fluorescent dyes (32). To generate the N-terminally SNAP-tagged human IP_3_R3 construct (SNAP-IP_3_R3), PCR was used to amplify an IP_3_R3 fragment (forward primer: CTCGGCGGTGGTTCTGGTGGTGGTTCTGGTATGAGTGAAATGTCCAGC, reverse primer: GAACCGCGGGCCCTCTAGATCAACCACTTTTGTACAAGAAAGCTGGGC), and *BsrGI* restriction digest was used to generate a backbone fragment. Gibson assembly was used to introduce a DNA string (GeneArt Strings, Thermo Fisher Scientific) encoding SNAPf (fast-labeling SNAP-tag) with a GGSGGGSG peptide linker between the backbone fragment and the IP_3_R3 fragment (Fig. 1*A*). For expression of SNAP-IP_3_R3 under control of a tetracycline-inducible promoter, SNAP-IP_3_R3 was transferred from pcDNA3.2 to pDONR221 and then to pT-REx-DEST30 using BP and LR Gateway cloning, respectively.

To generate the construct encoding IP_3_R3 with reduced affinity for IP_3_ (SNAP-IP_3_R3^RQ^, Fig. 6*A*), a DNA string was synthesized encoding a region of SNAP-IP_3_R3 between two restriction sites, *BsiWI* and *RsrII*, which contained an Arg to Gln mutation at residue 568 (R568Q, CGC to CAG). A *BsiWI/RsrII*-restriction digest of SNAP-IP_3_R3 in pDONR221 was used to generate a fragment encoding the remainder of the construct. Gibson assembly was used to assemble the two fragments, and SNAP-IP_3_R3^RQ^ was transferred to pT-REx-DEST30 using LR Gateway cloning. Sequences of all coding regions were confirmed using Sanger sequencing (Source BioScience). A silent point mutation was detected in bases (ATC to ATA) encoding Ile^2512^ in both constructs.

### Cell culture

HEK cells lacking all IP_3_R subtypes (HEK-3KO, #EUR030) or expressing only IP_3_R3 (HEK-IP_3_R3, IP_3_R1/2 knock-out, #EUR033) were generated by CRISPR/Cas9 (2) and obtained from Kerafast. HEK cells were cultured in Dulbecco’s modified Eagle’s medium/F-12 with Gluta-MAX (Thermo Fisher Scientific) supplemented with 10% fetal bovine serum (FBS, Sigma-Aldrich). Cells were maintained at 37 °C in humidified air with 5% CO_2_ and passaged every 3 to 4 days using Gibco TrypLE Express (Thermo Fisher Scientific). Regular screening confirmed that cells were free of *mycoplasma*.

To generate cells (HEK-SNAP-IP_3_R3 cells) expressing SNAP-IP_3_R under control of the Tet repressor (TetR), we used pcDNA6/TR, a plasmid encoding TetR, and the Invitrogen T-REx expression system. HEK-3KO cells were seeded in 25-cm^2^ flasks and after 24 h, when they were ∼70% confluent, they were transiently transfected using TransIT-LT1 Transfection Reagent (3 μl/μg DNA; Mirus Bio) with SNAP-IP_3_R3 in pT-REx-DEST30 (2.6 μg/T25 flask) and pcDNA6/TR (15.8 μg). After 24 and 48 h, medium was exchanged for fresh complete medium containing doxycycline (1 μg/ml) to block activity of TetR and derepress the expression of SNAP-IP_3_R3 (Fig. 1*B*).

### Labeling of cells with SNAP-tag

HEK-SNAP-IP_3_R3 cells were used 72 h after transfection for labeling of SNAP-IP_3_R3 with SNAP-Cell 647-SiR, a cell-permeable, far-red fluorescent label (excitation 645 nm; emission 661 nm). To avoid background labeling, it was essential to label cells in suspension before reattaching them to imaging dishes for microscopy (Fig. 1, *C–E*). Transfected cells were detached using TrypLE Express, resuspended in fresh complete medium (3 × 10^5^ cells in 300 μl) containing SNAP-Cell 647-SiR (1 μM), and incubated for 15 min at 37 °C with 5% CO_2_. Cells were then washed three times (650*g*, 2 min), resuspended in fresh complete medium, plated into a 35-mm glass-bottomed imaging dish (#1 cover glass) coated with 10 μg/ml human fibronectin, and used after 12 to 14 h (Fig. 1*B*).

### Fluorescence microscopy

Fluorescence microscopy used an inverted Olympus IX83 microscope equipped with a 100× oil-immersion TIRF objective (numerical aperture, NA = 1.49) or (for Fig. S1) a 60× oil-immersion objective (NA = 1.45). Illumination for TIRF microscopy was *via* a Cairn MultiLine LaserBank (488 and 647 nm) and an iLas2-targeted laser illumination system (Cairn Research), through which the excitation angle for TIRF was adjusted to achieve a theoretical penetration depth of 80 to 100 nm. Excitation light of 488 or 647 nm was passed through a quad-band filter set (TRF89902-EM, Chroma Technology), and emitted light was passed through an appropriate band-pass filter within a high-speed filter wheel (Cairn Optospin; peak/bandwidth: 525/50 or 700/75 nm). A 395-nm LED (SPECTRA X-light engine, Lumencor) was used for flash photolysis of ci-IP_3_. Detection of emitted light was *via* an EMCCD camera (iXon Ultra 897, Andor; 512 × 512 pixels; pixel size = 0.16 μm at 100× magnification). Image capture used MetaMorph Microscopy Automation and Image Analysis Software (version 7.10.1.161, Molecular Devices), through which image capture (control of shutters, etc.) was fully automated. Both live and fixed cells were imaged at 20 °C.

### Immunostaining

About 14 h after labeling, HEK-SNAP-IP_3_R3 cells in imaging dishes were fixed (20 min) with 4% paraformaldehyde (Alfa Aesar) in PBS (Thermo Fisher Scientific), washed twice in PBS, permeabilized (5 min) with Triton X-100 (0.25% in PBS, Thermo Fisher Scientific), washed twice in PBS, and blocked (1 h) in PBS with 5% bovine serum albumin (BSA; Europa Bioproducts). Cells were then incubated (12 h, 4 °C) with primary monoclonal mouse anti-IP_3_R3 antibody (Ab-IP_3_R3, 1:200, BD Biosciences, #610313, RRID: AB_397705) in PBS with 3% BSA, washed three times in PBS, and incubated (1 h, 20 °C) with secondary rabbit anti-mouse antibody conjugated to Alexa Fluor 488 (1:400, Thermo Fisher Scientific, #A11059, RRID: AB_2534106) in PBS with 3% BSA for 1 h. Cells were then washed five times in PBS before imaging. All steps were performed under conditions that minimized exposure to light. We confirmed the specificity of Ab-IP_3_R3 by demonstrating the absence of immunostaining in HEK-3KO cells (Fig. S1).

### Quantification of fluorescence images

All TIRF images were first background-corrected by subtracting the gray value of an area from outside a cell using MetaMorph or FIJI (https://fiji.sc/) (33). To quantify the expression of SNAP-IP_3_R3, fluorescence (647 nm) was measured as the mean gray value from a region (19.2 × 19.2 μm) within which Ca^2+^ puffs were recorded. We refer to this measure of SNAP-IP_3_R3 expression as SNAP-647 fluorescence from ROI^puffs^. To allow comparisons between experiments, fluorescence (F_TetraSpeck_) from TetraSpeck beads (Thermo Fisher Scientific, excitation 660 nm, emission 680 nm) was recorded and used to normalize measurements of SNAP-IP_3_R3 fluorescence (F_SNAP_). For experiments addressing the effects of varying SNAP-IP_3_R3 expression (Figure 4, Figure 5, Figure 6, Figure 7, Figure 8), normalized values (10, 000 × F_SNAP_/F_TetraSpeck_) were used for quantitative analyses.

For comparisons between IP_3_R3 immunostaining (488 nm) and SNAP-IP_3_R3 labeling (647 nm) (Fig. 2*C*), the mean gray value from a ROI demarcating the entire TIRF footprint of the cell was measured. Pixel-based colocalization analysis used the FIJI plugin JACoP (34). Images were manually thresholded before computing Manders’ split coefficients (M); M1 reports the fraction of IP_3_R3 immunofluorescence coinciding with SNAP-IP_3_R3 fluorescence, and M2 reports the fraction of SNAP fluorescence coincident with immunofluorescence (Fig. 2*B*). M values of 1.0 indicate perfect colocalization. Costes’ randomizations (100 iterations, pixel-block size = 5) were generated for each image and used to assess the statistical significance of any colocalization (35).

For fluorescence intensity measurements of individual puncta, ROI (19.2 × 19.2 μm) were background-subtracted (rolling ball = 50 pixel) in FIJI to improve signal-to-noise. The FIJI plugin TrackMate (v 6.0.1) was used for automated detection of IP_3_R3 immunofluorescent puncta (Fig. 2*F*) or SNAP-IP_3_R3 puncta (Fig. 5, *B* and *C*) (36).

Within TrackMate, we used an estimated punctum size of 600 nm for spot detection and applied a Laplacian of Gaussian filter to detect spot maxima with sub-pixel accuracy. Briefly, the Gaussian filter smooths noise, while the Laplacian filter is used to identify edges (36). Detected spots were filtered by ‘Quality’ until only genuine puncta (confirmed by manual inspection) were detected; the selection criteria require a punctum to both exceed a critical fluorescence intensity and to fall below a critical size (diameter < 600 nm). To compare fluorescence from immunostaining and SNAP-tag labeling (Fig. 2*F*), the coordinates of the pixel with the maximum fluorescence intensity for each detected spot in the IP_3_R3 immunofluorescence image were mapped onto the corresponding pixel of the SNAP-IP_3_R3 image. We used these point fluorescence intensity measurements to compare expression determined by immunostaining and SNAP-tag labeling. The approach is a valid measure of the fluorescence intensity of the entire punctum because the point intensity is linearly related to total intensity of the spot for the IP_3_R3 immunofluorescence image (Fig. S2).

### High-resolution imaging of Ca^2+^ puffs

To allow imaging of Ca^2+^ puffs, cells were loaded with a Ca^2+^ indicator (Cal520), EGTA to restrict Ca^2+^ diffusion (4), and ci-IP_3_ to allow controlled release of intracellular i-IP_3_ by photolysis. The slow on-rate for Ca^2+^ binding to EGTA is sufficient to allow EGTA to buffer Ca^2+^ diffusing between Ca^2+^ puff sites, which are typically a few μm apart. EGTA thereby restrains propagation of global Ca^2+^ signals, but the on-rate is too slow for EGTA to intercept Ca^2+^ diffusing between IP_3_Rs within sites (200–400 nm) (4, 5, 6, 7). EGTA does not, therefore, significantly affect the properties of individual Ca^2+^ puffs (4, 37, 38). HEK-SNAP-IP_3_R3 cells in imaging dishes were washed with Hepes-buffered saline (HBS: 135 mM NaCl, 5.9 mM KCl, 1.2 mM MgCl_2_, 1.5 mM CaCl_2_, 11.5 mM glucose, 11.6 mM Hepes, pH 7.3; Merck), incubated (1 h) with Cal520-AM (5 μM) and ci-IP_3_/PM (1 μM) in HBS with pluronic acid (0.02%, v/v). Cells were then washed and incubated with EGTA-AM (5 μM) in HBS with pluronic acid (0.02%). After 45 min, cells were washed and incubated in HBS for a further 30 min before imaging. All steps were performed at 20 °C with minimal exposure to light.

TIRF microscopy was used to record Cal520 fluorescence (488 nm) within ∼100 nm of the plasma membrane. To achieve the high temporal resolution required to resolve the kinetics of Ca^2+^ puffs, image streams were captured at 200 frames s^-1^ (5-ms intervals) from a 19.2 × 19.2 μm ROI (∼25–50% of the TIRF footprint of a cell, referred to as ROI^puffs^). Image stacks typically comprised 6000 to 10,000 frames (∼30–50 s). After 2 s of recording, a UV flash lasting 250 ms (25 ms in parts of Fig. 8) was delivered uniformly to the entire field to uncage ci-IP_3_. A 50:50 mirror in the light path allowed simultaneous excitation by the 488-nm laser and 395-nm LED. Image stacks were streamed directly to RAM in MetaMorph and then exported as.tif files.

### Detection and analysis of Ca^2+^ puffs

Raw image stacks were background-corrected in MetaMorph and frames containing the UV flash artefact were removed in FIJI. Automated detection and analysis of Ca^2+^ puffs were performed using the detect_puffs plugin in FLIKA, an open-source Python-based image processing and analysis software (39, 40). An F/F_0_ stack was generated by dividing the fluorescence intensity of each pixel from each frame (F) by the average intensity of that pixel over the first 390 frames before photorelease of i-IP_3_ (F_0_). Image stacks (F/F_0_) were then divided by the SD of the baseline F/F_0_ images, a Gaussian blur (σ = 2) was applied, and these stacks were then used to identify Ca^2+^ puffs and measure their properties.

When a global increase in fluorescence obscured Ca^2+^ puffs, a Butterworth temporal bandpass filter (low frequency cut-off = 0.01) was applied to remove the slow increase in baseline fluorescence. In some cases, global increases in [Ca^2+^]_c_ completely obscured Ca^2+^ puffs causing premature termination of the analysis; the shortest of these terminated recordings was ∼12 s. The recording interval was defined as the time from the first Ca^2+^ puff to either the end of the recording or the time when it became impracticable to resolve Ca^2+^ puffs against an elevated global [Ca^2+^]_c_.

FLIKA uses a threshold-cluster algorithm (41) to identify pixels brighter than a user-defined threshold (0.25 for our analysis). The brightest pixels are considered cluster centers, and adjacent bright pixels are included in the cluster. Each cluster represents a Ca^2+^ puff, to which a 2D Gaussian is fitted to identify its centroid. The centroid, defined with sub-pixel resolution, reports the center-of-mass of the clustered IP_3_Rs underlying the Ca^2+^ puff (37). The centroids of all Ca^2+^ puffs were mapped onto the TIRF footprint of the cell. To measure the amplitudes and kinetics of individual Ca^2+^ puffs, an F/F_0_ trace was produced from a small ROI (1.76 × 1.76 μm) centered on the centroid of the Ca^2+^ puff. Detected puffs were manually inspected to exclude noisy traces and to ensure the entire F/F_0_ trace was included in the analysis. In some cases, ‘square’ puffs (detected by eye) were manually removed from analyses of kinetic properties (Fig. S6). Files containing puff properties generated by FLIKA were exported to Microsoft Excel for further analysis.

### Identification of Ca^2+^ puff sites

Ca^2+^ puffs recur at the same stable sites over many minutes, reflecting Ca^2+^ release from immobile clusters of IP_3_Rs (7, 8). In our analyses, Ca^2+^ puffs that lie within 1 μm (6.25 pixels) of each other were considered to belong to the same Ca^2+^ puff site. FLIKA successfully identifies these sites when Ca^2+^ puffs are relatively infrequent and sparsely distributed. However, when Ca^2+^ puffs are more frequent and more densely packed, FLIKA merges large numbers of Ca^2+^ puffs into fewer sites, causing the number of sites to be underestimated (Fig. S5, *A* and *B*). This occurs because FLIKA assigns Ca^2+^ puffs to the same site if each successively assessed Ca^2+^ puff is < 1 μm from the last; hence, a site can grow in a chain-like fashion as densely packed Ca^2+^ puff centroids become linked across considerable distances. To overcome this difficulty, we used a MATLAB script, ClusterXYpoints (version 1.5.0.0, https://www.mathworks.com/matlabcentral/fileexchange/56150-distance-based-clustering-of-a-set-of-xy-coordinates) (42), to identify Ca^2+^ puff sites. The XY coordinates of Ca^2+^ puff centroids generated by FLIKA were saved as .csv files and imported into MATLAB (version R2021a). ClusterXYpoints starts by sorting the XY coordinates of Ca^2+^ puffs (a.k.a. points) in ascending order. The first point is assigned as a cluster, and the cluster centroid is calculated. If the second point is < 1 μm from the first cluster centroid, it is added to the cluster and the centroid is recalculated. Subsequent points are added to the cluster if they are < 1 μm from the updated centroid of the cluster. If a point is > 1 μm from the current cluster centroid, a new cluster centroid is generated. The process continues until all Ca^2+^ puffs have been assigned to a site.

ClusterXYpoints detected the same sites as FLIKA when Ca^2+^ puffs were infrequent, but it identified more sites than FLIKA when Ca^2+^ puffs were more frequent. At the highest frequencies of Ca^2+^ puffs, when FLIKA merged most Ca^2+^ puffs into a single site, ClusterXYpoints continued to identify discrete sites (Fig. S5, *A* and *B*). We therefore used ClusterXYpoints to identify the number of Ca^2+^ puff sites. The problems encountered with ‘coalescing’ Ca^2+^ puff sites in FLIKA analyses arise only because the centroids of many temporally separated Ca^2+^ puffs are overlaid for analysis, they therefore have no impact on the analyses of individual Ca^2+^ puffs.

### Epifluorescence imaging of cell populations

For measuring SNAP-IP_3_R3 expression in cell populations (Fig. 1*E*), HEK-SNAP-IP_3_R3 or mock-transfected HEK-3KO cells were labeled with SNAP-Cell 647-SiR. Cells were washed with HBS and loaded with Calbryte590-AM (2 μM) in HBS with pluronic acid (0.02%) for 1 h at 20 °C in darkness. Here, Calbryte590 is used to define cell boundaries rather than as a Ca^2+^ indicator. Cells were washed and incubated in HBS for a further 1 h before imaging.

Epifluorescence imaging used an EVOS M7000 Cell Imaging System equipped with a 10× objective and a CMOS camera with a large field of view (Thermo Fisher Scientific). Calbryte590 and SNAP-Cell 647-SiR fluorescence were captured using EVOS LED cubes for Texas Red and Cy5, respectively. For image analysis, Calbryte590 fluorescence was used to demarcate whole cells because it is bright and uniformly distributed in the cytosol, making it ideal for automated segmentation. A custom-written ImageJ macro was used for segmentation (available from H.A.S.); the detected cell boundaries were then confirmed by manual inspection. Raw mean SNAP-IP_3_R3 fluorescence intensity was measured from individual whole-cell ROIs.

### Statistical analyses

Most data are presented as mean ± SD or S.E.M. Numbers of cells, Ca^2+^ puffs, and independent analyses are reported in figure legends. Statistical comparisons used unpaired Student’s *t* tests; correlation analyses used Pearson’s correlation coefficient; comparisons of frequency distributions used χ^2^ test for trend (GraphPad Prism 5, GraphPad). The statistical significance of colocalization analyses (Manders’ split coefficients) was determined after Costes’ randomization, where a Costes’ *p* value > 0.95 indicates that < 0.05 of the randomized images had a Pearson’s correlation coefficient value greater than the nonrandomized image (*i.e.*, *p* < 0.05) (35).

## Data availability

All data required to support the conclusions presented are contained with the article.

## Supporting information

This article contains supporting information (Figs. S1–S6) (39, 40).

## Conflict of interest

The authors declare that they have no conflicts of interest with the contents of this article.
