# Supplementary material for: Dissociation of inositol 1,4,5-trisphosphate from IP3 receptors contributes to termination of Ca2+ puffs
Source: J Biol Chem. 2023 Jan 5;299(2):102871. doi: 10.1016/j.jbc.2023.102871 (PMC9971896; doi:10.1016/j.jbc.2023.102871)
Supplement: Supplementary figures [file mmc1.pdf]

Figure S1

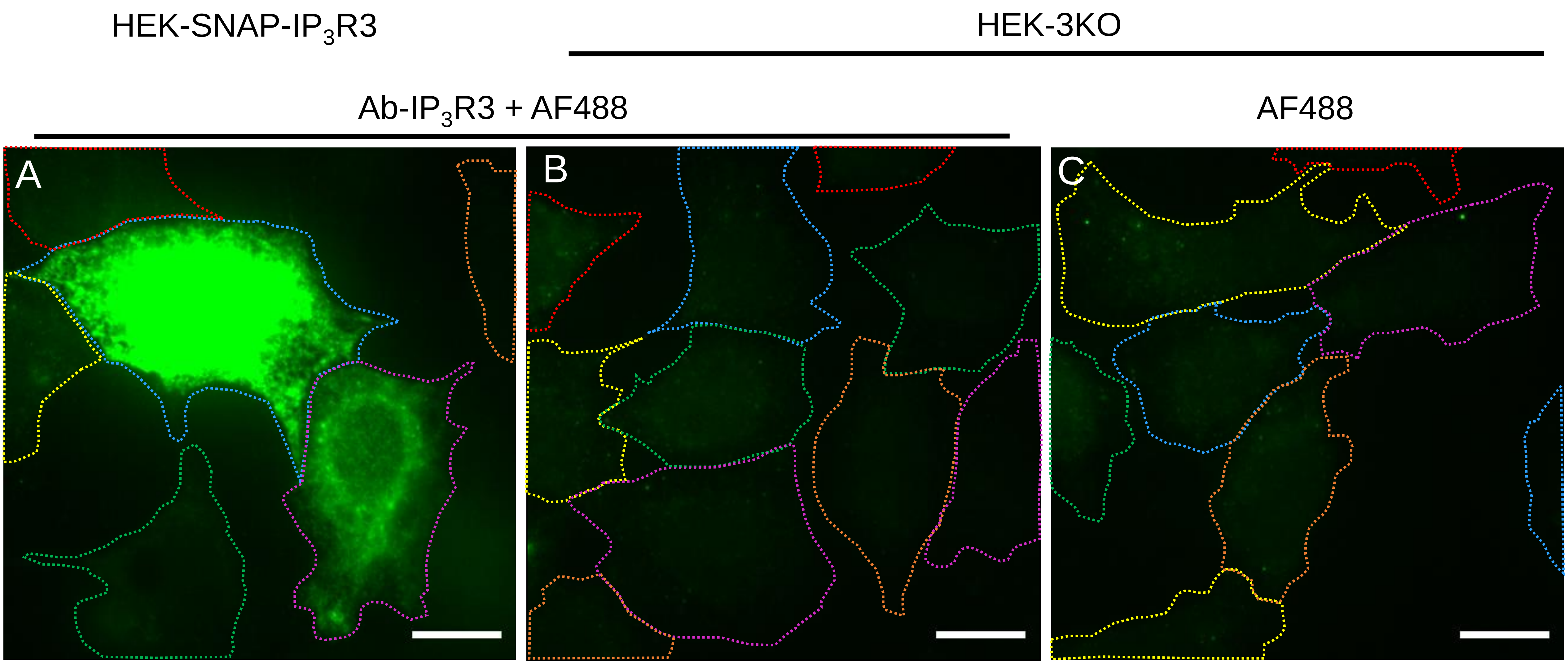

Figure S1. Ab-IP$_3$R3 selectively recognizes IP$_3$R3 in immunocytochemical analyses. *A-C*, Typical epifluorescence images of HEK-SNAP-IP$_3$R3 cells (*A*) or HEK-3KO cells (*B*, *C*) stained with Ab-IP$_3$R3 and Alexa Fluor 488-conjugated secondary antibody (AF488) (*A*, *B*), or with only AF488 (*C*). Images were captured under identical conditions and with similar display values. Scale bars represent 20 µm. Cell borders are indicated by dashed lines. Results, typical of two independent analyses, demonstrate that Ab-IP$_3$R3 selectively recognizes IP$_3$R3.

Figure S2

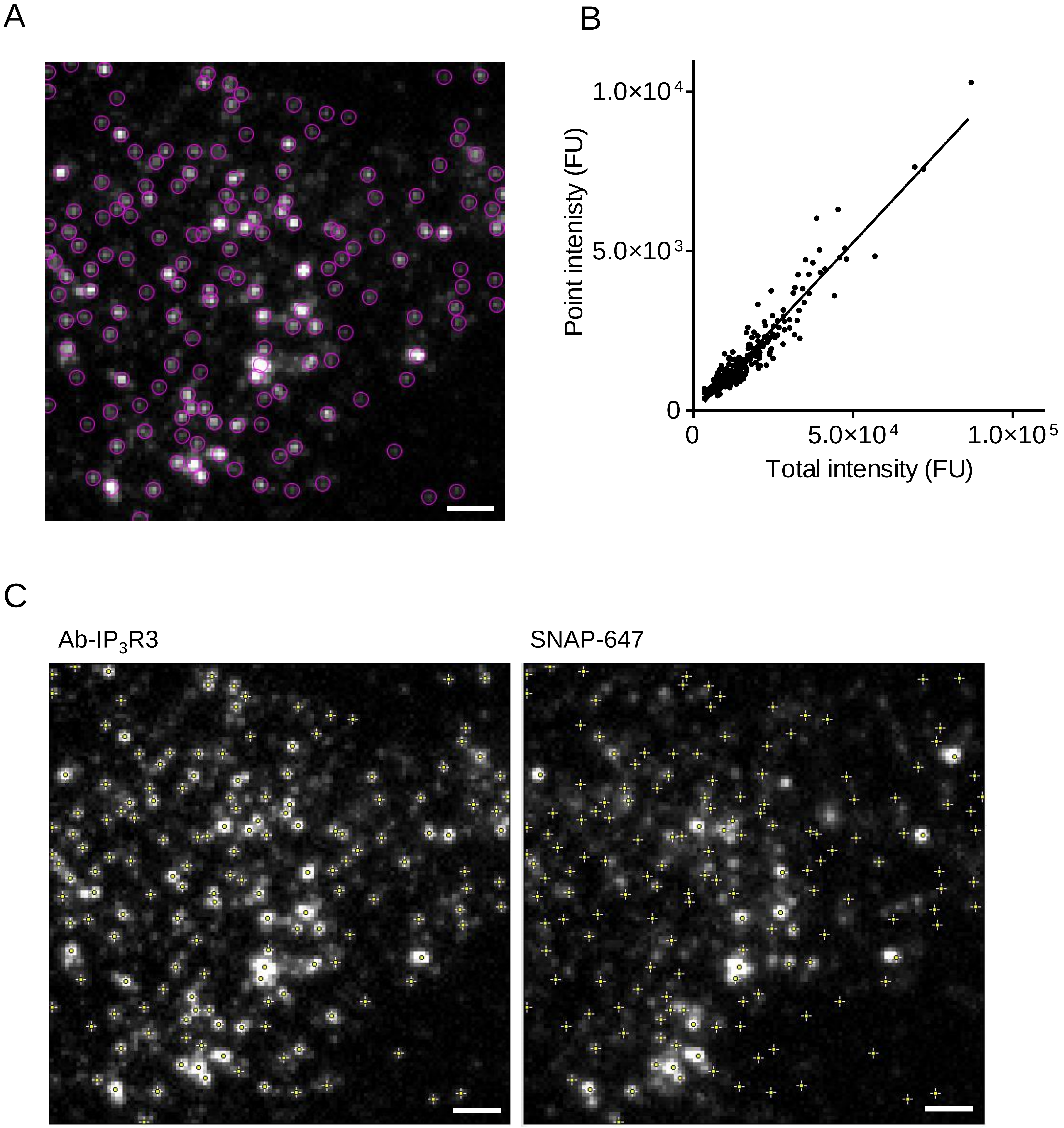
A
B
1.0×10⁴
5.0×10³
0
Point intenisty (FU)
0
5.0×10⁴
1.0×10⁵
Total intensity (FU)
C
Ab-IP3R3
SNAP-647

Figure S2

Figure S2. Use of single-pixel measurements to compare fluorescence intensities of SNAP-IP_3R3 puncta identified with Ab-IP_3R3 or SNAP-647. *A*, HEK-SNAP-IP_3R3 cells were immunostained with Ab-IP_3R3, and puncta within the TIRF field were identified using TrackMate (purple circles). *B*, Analyses of relationships between the fluorescence intensities of SNAP-647 and Ab-IP_3R3 puncta (**Fig. 2*F***) require transfer of quantified fluorescence intensities between images, but it is impossible to transfer ROI identified by TrackMate between images. Hence, it was important to establish whether the fluorescence intensity measured at the brightest pixel within each ROI ('point intensity') provides a reliable surrogate measure of the total fluorescence of the ROI. Results from a single cell immunostained with Ab-IP_3R3 show the linear relationship between the total fluorescence intensity of each ROI and its corresponding point intensity. The results establish the linear correlation between total and point fluorescence; Pearson correlation coefficient, $r = 0.95$, $p < 0.0001$. These observations validate our use of point intensities to compare puncta between images. *C*, Point ROI (crosses) from images of Ab-IP_3R3 and SNAP-IP_3R3 were compared to allow the point intensity of each punctum for each label to be plotted against each other (**Fig. 2*F***). Scale bars (*A* and *C*) represent 2 µm.

Figure S3

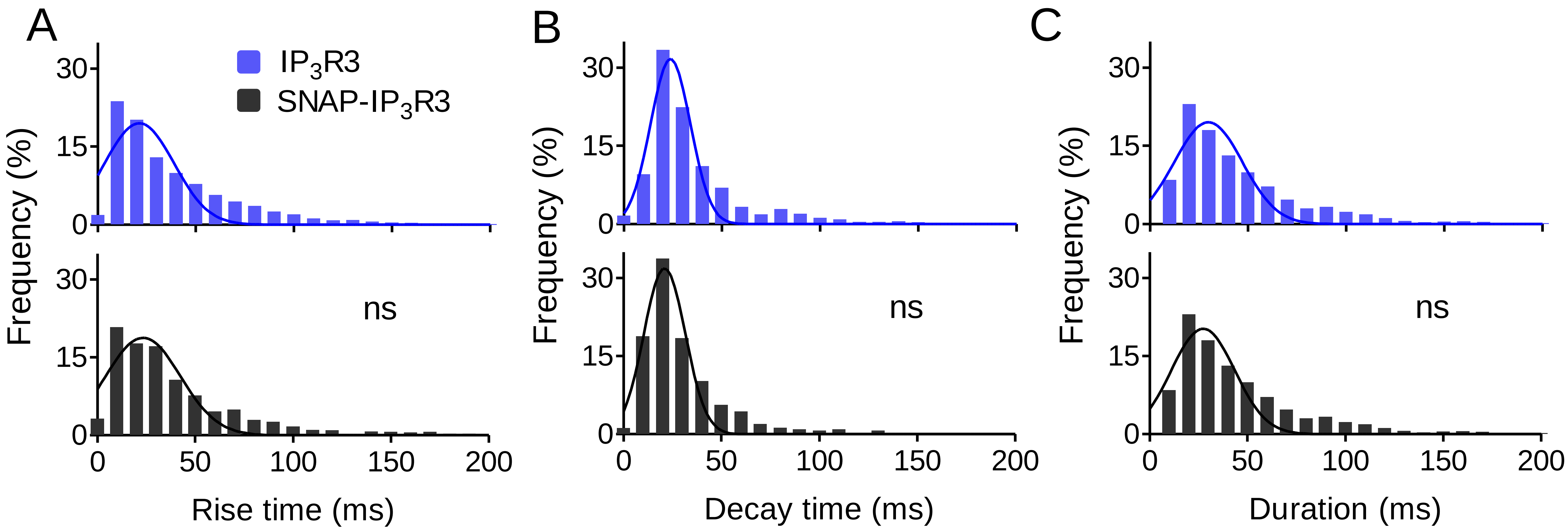

Figure S3. Frequency distributions of the kinetic properties of Ca^{2+} puffs for IP_3R3 and SNAP-IP_3R3 are indistinguishable. *A*-*C*, Frequency distributions with Gaussian curve-fits for rise times (*A*), decay times (*B*) and durations (*C*) of individual Ca^{2+} puffs evoked by photolysis of ci-IP_3 in HEK-IP_3R3 or HEK-SNAP-IP_3R3 cells. ns $p > 0.05$, χ^2 test for trend. Results are from 1653 puffs in 19 cells from 5 experiments (IP_3R3) and 1219 puffs in 18 cells from 3 experiments (SNAP-IP_3R3). Ca^{2+} puffs with 'square' temporal profiles (**Fig. S6**) were not excluded from these analyses. Summary results shown in **Fig. 3*G*.**

Figure S4

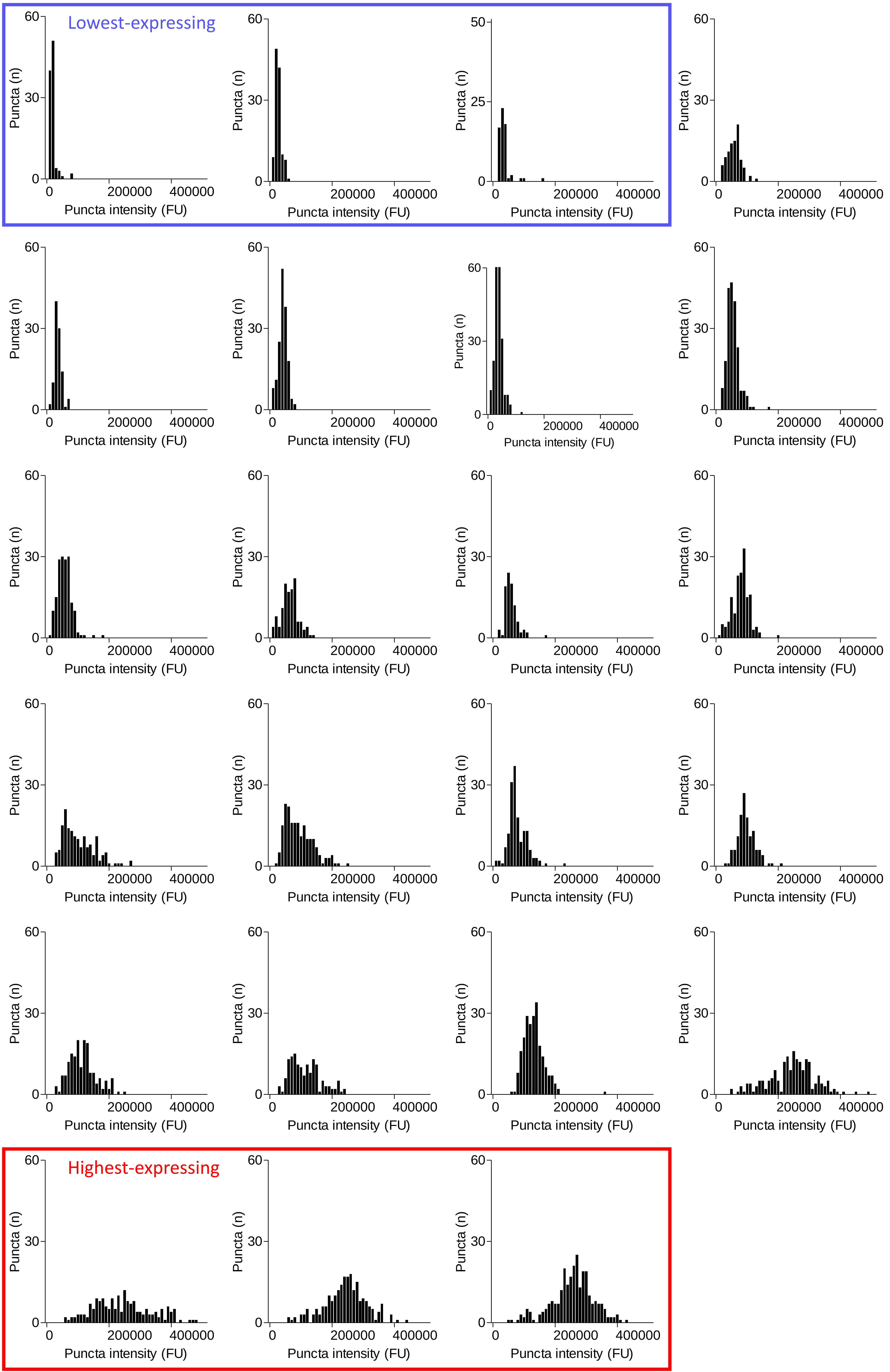
Lowest-expressing
Highest-expressing
Puncta (n)
Puncta intensity (FU)
0
200000
400000
60
30
50
25

Figure S4

Figure S4. Fluorescence intensity distributions for individual SNAP-IP$_3$R3 puncta from cells expressing different numbers of SNAP-IP$_3$R3. SNAP-647 fluorescence intensity distributions for individual puncta from all cells shown in **Fig. 5*C***. Plots are ordered from lowest (top) to highest SNAP-IP$_3$R3 expression (measured as SNAP-647 fluorescence from ROI^{puffs}). Cells used for the analysis in **Fig. 5*D*** are enclosed by blue and red boxes. As SNAP-IP$_3$R3 expression increases, the numbers of puncta increase and the distributions of fluorescence intensities for individual puncta shift rightward. The results indicate that as SNAP-IP$_3$R3 expression increases, SNAP-IP$_3$R3 form more clusters and each contains more SNAP-IP$_3$R3.

Figure S5

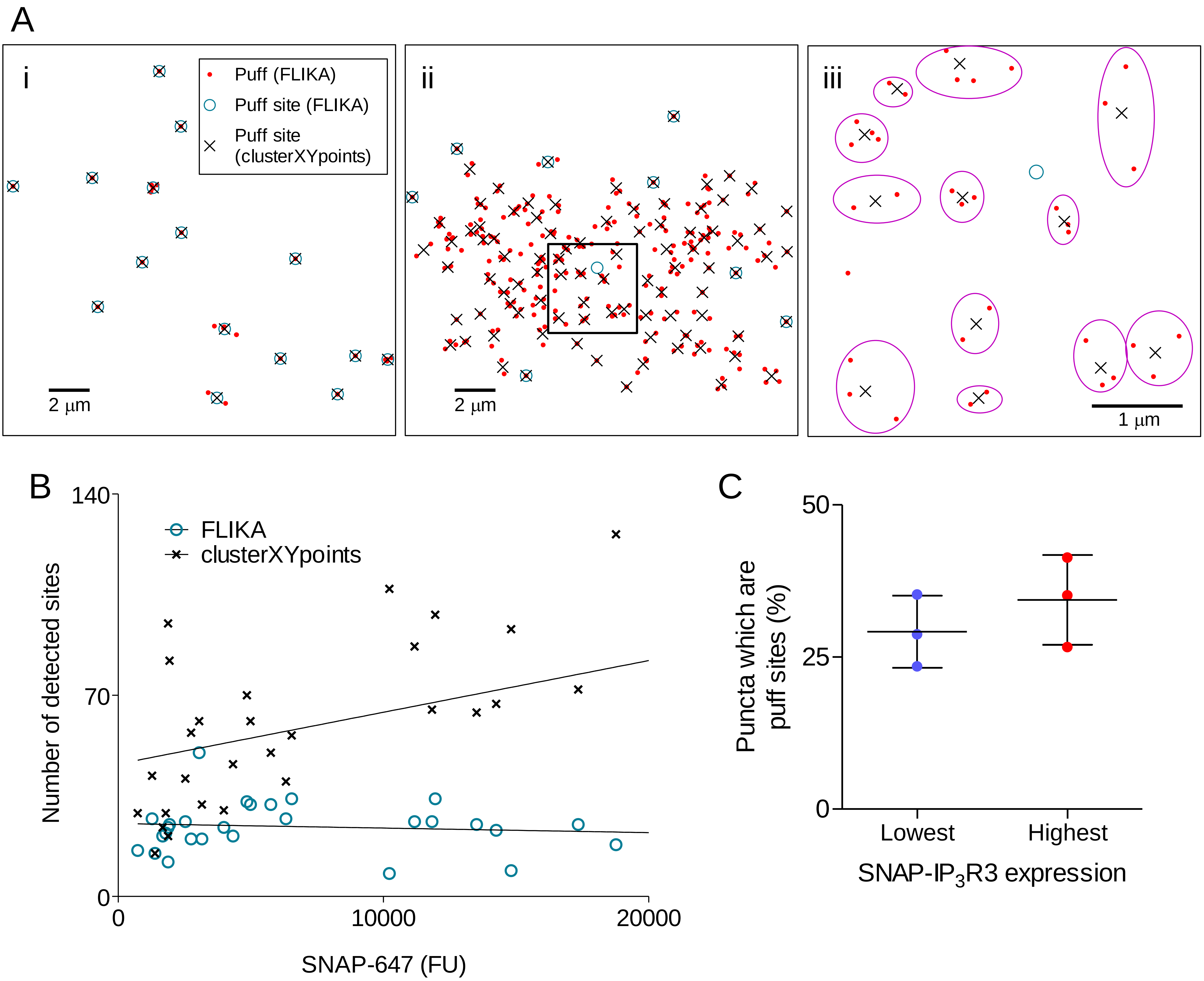
A
i
Puff (FLIKA)
Puff site (FLIKA)
Puff site (clusterXYpoints)
2 μm
ii
2 μm
iii
1 μm
B
Number of detected sites
140
70
0
FLIKA
clusterXYpoints
0
10000
20000
SNAP-647 (FU)
C
Puncta which are puff sites (%)
50
25
0
Lowest
Highest
SNAP-IP3R3 expression

Figure S5

Figure S5. Identification of Ca^{2+} puff sites. FLIKA (39, 40) reliably allocates Ca^{2+} puffs to sites when Ca^{2+} puffs occur at moderate frequency, but when Ca^{2+} puffs are very frequent it tends to merge Ca^{2+} puffs into 'mega-sites' that under-estimate the likely number of sites. We therefore had to consider alternative approaches to allocating Ca^{2+} puffs to sites. *A*, Coordinates of Ca^{2+} puffs and Ca^{2+} puff sites mapped onto the ROI^{puffs} of cells expressing low (*i*) or high (*ii*) levels of SNAP-IP_3R3. Red points indicate the centroid of a detected puff. Blue circles indicate the centroid of a puff site detected in FLIKA (where puffs are grouped based on the distance to a neighboring puff). Black crosses indicate centroid of a puff site detected using ClusterXYpoints (puffs grouped based on distance to centroid). Panel *iii* is an enlargement of the boxed area in *ii*, with purple circles showing puffs grouped into sites by ClusterXYpoints that would have erroneously been identified as a single group by FLIKA at high puff densities (all Ca^{2+} puffs at the center of the field are assigned to a single site, blue circle). *B*, Number of detected puff sites plotted against SNAP-IP_3R3 expression level for both algorithms. Since FLIKA under-estimates the number of puff sites when puffs are very frequent, we used ClusterXYpoints, rather than FLIKA, to assign Ca^{2+} puffs to sites. *C*, The number of Ca^{2+} puff sites detected using ClusterXYpoints relative to the number of SNAP-IP_3R3 puncta detected using TrackMate from the cells highlighted in **Fig. S4** were used to estimate the percentage of puncta expected to be active Ca^{2+} puff sites. Results show individual values from 3 cells, mean ± S.D..

Figure S6

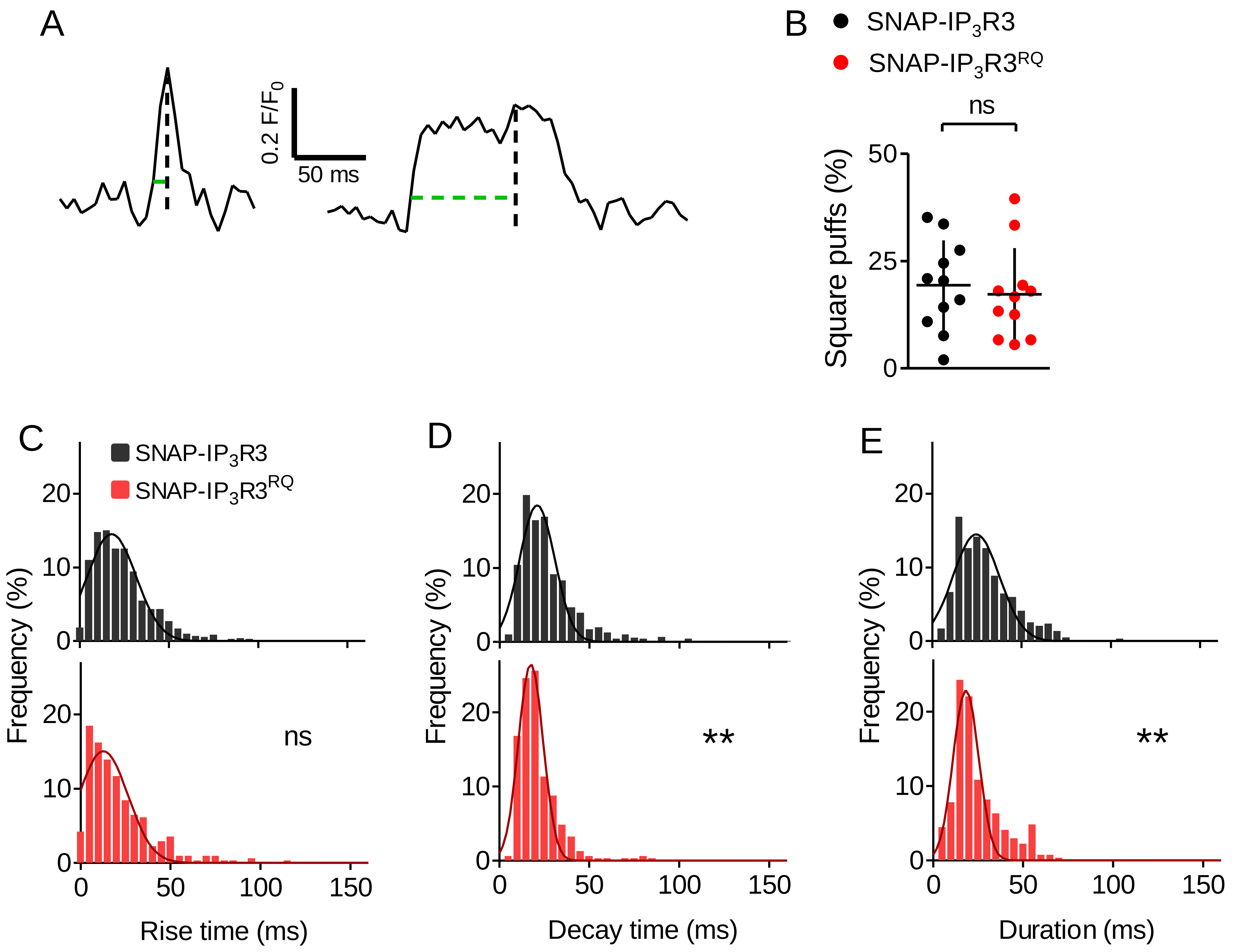

Figure S6. Unbiased exclusion of 'square' Ca^{2+} puffs for comparisons of kinetic properties for SNAP-IP_3R3 and SNAP-IP_3R3^{RQ}. *A*, Examples of 'typical' and 'square' Ca^{2+} puffs evoked by photolysis of ci-IP_3 in a HEK-SNAP-IP_3R3^{RQ} cell. Puffs with similar profiles were observed in HEK-SNAP-IP_3R3 and HEK-IP_3R3 cells. Peak amplitude (black dotted line) and rise time (green dotted line) are measured between the indicated points in the FLIKA algorithm. Flickering fluorescence intensities around the sustained plateau of 'square' Ca^{2+} puffs cause erratic prolongation of the time to peak and correspondingly erratic overestimates of rise times. Our comparisons of the kinetics of individual Ca^{2+} puffs for SNAP-IP_3R3 and SNAP-IP_3R3^{RQ} excluded 'square' events. *B*, Similar fractions of 'square' events were identified and excluded from the analysis of Ca^{2+} puffs evoked by SNAP-IP_3R3 and SNAP-IP_3R3^{RQ} (**Fig. 7 and 8**), demonstrating that the exclusions did not bias the analyses. ns $p > 0.05$, unpaired Student's *t* test. *C-E*, Frequency distributions with Gaussian curve-fits for rise times (*C*), decay times (*D*) and durations (*E*) of individual Ca^{2+} puffs evoked by SNAP-IP_3R3 or SNAP-IP_3R3^{RQ}. ns $p > 0.05$, ** $p < 0.01$, χ^2 test for trend. Results (*B-E*) from 711 Ca^{2+} puffs in 11 cells from 4 experiments (SNAP-IP_3R3) and 309 puffs in 11 cells from 5 experiments (SNAP-IP_3R3^{RQ}).
